# Diagnostic and therapeutic pathways for lymphoma patients: expert consensus through Nominal Group Technique and Delphi methodology

**DOI:** 10.3389/fonc.2025.1627175

**Published:** 2025-08-13

**Authors:** Attilio Guarini, Valentina Bozzoli, Sabino Ciavarella, Michele Cimminiello, Francesca Donatelli, Angelo Fama, Vincenza Fernanda Fesce, Vincenzo Fraticelli, Francesco Gaudio, Giuseppina Greco, Augusto Martellini, Francesca Merchionne, Rosanna Maria Miccolis, Carla Minoia, Elsa Pennese, Tommasina Perrone, Potito Rosario Scalzulli, Anna Scattone, Tetiana Skrypets, Mariarosaria Specchia, Lorenzo Tonialini, Mariarosaria Valvano, Vincenzo Pavone

**Affiliations:** ^1^ Hematology Unit, IRCCS Istituto Tumori “Giovanni Paolo II”, Bari, Italy; ^2^ UO Ematologia e Trapianto di Cellule Staminali, Ospedale Vito Fazzi, asl Lecce, Lecce, Italy; ^3^ SIC di Ematologia con TMO, AOR San Carlo di Potenza, Potenza, Italy; ^4^ UOC Ematologia e Trapianto di CSE azienda ospedaliera C.Panico Tricase, Lecce, Italy; ^5^ UOS Ematologia, Dipartimento di Medicina Interna e Specialità Mediche, Ospedale “Giuseppe Mazzini” Hospital, ASL Teramo, Teramo, Italy; ^6^ UO Ematologia con Trapianto di CSE Azienda Ospedaliero, Universitaria Policlinico Riuniti di Foggia, Foggia, Italy; ^7^ Unità Operativa Semplice a valenza Dipartimentale di Onco-Ematologia, Campobasso, Italy; ^8^ Unit of Hematology, “F. Miulli” University Hospital, Bari, Italy; ^9^ Department of Medicine and Surgery, LUM University “Giuseppe Degennaro”, Bari, Italy; ^10^ Polistudium SRL, Milan, Italy; ^11^ U.O. Ematologia Ospedale “Antonio Perrino”, Brindisi, Italy; ^12^ UOC Ematologia con Trapianto P.O. “Mons.Dimiccoli”, Barletta, Italy; ^13^ UOC Ematologia Clinica Dipartimento Oncologico Ematologico Presidio Ospedaliero Spirito Santo, Pescara, Italy; ^14^ Hematology and Stem Cells Transplantation, AOUC Policlinico, Bari, Italy; ^15^ UOC Ematologia e Trapianto di Cellule Staminali Emopoietiche, Fondazione IRCCS “Casa Sollievo della Sofferenza”, San Giovanni Rotondo, FG, Italy; ^16^ Pathology Unit, IRCCS Istituto Tumori “Giovanni Paolo II”, Bari, Italy; ^17^ S.C. Ematologia e Trapianto di CSE, Ospedale “S. G. Moscati” ASL Taranto, Taranto, Italy

**Keywords:** lymphoma diagnosis and treatment, NGT, Delphi consensus, advanced molecular diagnostics, multidisciplinary cancer care, immunotherapy, CAR-T therapy

## Abstract

**Introduction:**

Lymphomas encompass a heterogeneous group of B- and T-cell malignancies necessitating a complex and precise management. With the aim to define standardized diagnostic and therapeutic pathways across multiple hematology centers in Italy, the “Right Path 4 Lymphomas” project was designed as a multidisciplinary expert platform designed to establish consensus-driven diagnostic and therapeutic pathways.

**Methods:**

Using a two-phase methodology – the Nominal Group Technique followed by a Delphi process – experts systematically evaluated and prioritized key diagnostic and therapeutic topics for five major lymphoma subtypes: classical Hodgkin lymphoma, diffuse large B-cell lymphoma, follicular lymphoma, mantle cell lymphoma, and peripheral T-cell lymphomas.

**Results:**

The Delphi process achieved a high level of consensus on 264 of 270 statements (97.8%), reinforcing the importance of multidisciplinary collaboration in shaping evidence-based recommendations. Key areas of agreement included histopathologic and molecular diagnostic standards, risk-adapted treatment approaches integrating novel immunotherapies, and structured follow-up strategies. However, areas of debate remained over the clinical utility of minimal residual disease monitoring, optimal sequencing of immunotherapies, and the potential of CAR-T therapy versus bispecific antibodies.

**Discussion:**

This project highlights the need for a structured, consensus-driven approach to lymphoma care that aligns with evolving international guidelines while addressing the distinct regulatory and healthcare landscape in Italy. The findings provide a valuable framework for clinicians and policymakers to optimize lymphoma management, balancing innovation with the allocation of resources and clinical feasibility.

## 1 Introduction

Lymphomas constitute a heterogeneous group of neoplasms of B- or T-cell origin, accounting for approximately 5% of all cancers worldwide (1, 2). Their incidence varies significantly across different regions, with non-Hodgkin lymphomas (NHLs) representing the most prevalent hematologic malignancy, comprising nearly 3% of all cancer diagnoses (3, 4). Lymphomas can develop at any age and exhibit diverse clinical behaviors, depending on the histologic subtype (1, 2, 5).

The 2022 World Health Organization (WHO) and 2022 International Consensus Classification (ICC) classifications have integrated traditional histopathological features with recent molecular discoveries, refining diagnostic precision and therapeutic decision-making (6, 7). Advances in molecular research have deepened the understanding of lymphoma pathophysiology, leading to the identification of novel therapeutic targets and mechanisms. Over the past decade, these insights have led to the clinical implementation of previously experimental targeted therapies (2). As a result, lymphoma patients now benefit from an expanding array of treatment options, which have contributed to improved disease-free survival and overall survival (OS), particularly in well-characterized, frequently studied histologic subtypes.

Significant therapeutic innovations in lymphoma treatment in recent years include highly targeted immunotherapies, such as bispecific T-cell engagers (BiTEs) and chimeric antigen receptor T-cell (CAR-T) therapy (8, 9). Additionally, novel monoclonal antibodies – both conjugated (e.g., brentuximab vedotin, polatuzumab vedotin, loncastuximab tesirine) and non-conjugated (e.g., immune checkpoint inhibitors such as nivolumab and pembrolizumab, as well as tafasitamab and obinutuzumab)—have expanded the therapeutic landscape (10, 11). Small-molecule inhibitors, including anti-BRAF agents, covalent Bruton tyrosine kinase inhibitors (BTKi; ibrutinib, acalabrutinib, zanubrutinib), and the non-covalent BTKi pirtobrutinib, have further enhanced treatment precision (12). Recently approved in Italy for various lymphoma subtypes, these therapies are available as monotherapies or combination regimens.

Concurrent advancements in diagnostic technologies have significantly improved the precision of disease classification and treatment selection. The integration of next-generation sequencing (NGS), NanoString, and fluorescence *in situ* hybridization (FISH) into routine diagnostics has refined molecular profiling, thus enabling personalized therapy selection (13). Additionally, imaging modalities such as positron emission tomography/computed tomography (PET/CT), along with semi-quantitative parameters, such as total metabolic tumor volume and the Deauville score (DS), have facilitated more accurate assessment of disease burden and treatment response, supporting tailored therapeutic strategies and early identification of refractory patients (14). These technological advancements have enhanced risk stratification, enabling innovative, integrative therapeutic approaches for high-risk patients.

Given the rapid evolution of lymphoma research and treatment, regular multidisciplinary discussions among specialists – within scientific societies and regional working groups – are essential for optimizing diagnostic and therapeutic strategies. In this context, the “Right Path 4 Lymphomas” project was conceived as a structured expert platform designed to establish consensus-driven diagnostic and therapeutic pathways (DTPs). The initiative aimed to harmonize clinical practice across multiple hematology centers in four Italian regions, ensuring standardized, evidence-based patient care. The importance of structured DTPs is particularly evident in complex diseases such as lymphoma, where diagnostic accuracy and treatment efficacy are paramount.

Beyond standardization of clinical practice, “Right Path 4 Lymphomas” was designed to balance clinical efficacy with resource availability, providing a clear framework for diagnostics and therapeutics. This structured approach ensures consistency in care delivery, minimizes variability in clinical practice, and optimizes healthcare resource utilization. It offers clinicians standardized, evidence-based protocols integrating the latest advances in molecular diagnostics and treatment. At the same time, it guarantees patients access to the best available care within the constraints of the healthcare system.

To achieve its objectives, “Right Path 4 Lymphomas” employed a rigorous two-phase methodology: the Nominal Group Technique (NGT) followed by a Delphi process. The NGT phase facilitated the generation and prioritization of key statements, while the Delphi process enabled experts to reach consensus on diagnostic and therapeutic management across five selected lymphoma subtypes. This methodology synthesizes multidisciplinary expertise, providing a comprehensive, expert-driven roadmap for enhancing lymphoma care in both research and clinical settings.

## 2 Methods

This project, called “Right Path 4 Lymphomas,” employed a two-phase methodology, combining the NGT with a Delphi process to systematically collect expert opinions and achieve consensus on diagnostic and therapeutic approaches in lymphoma management. This structured methodology facilitated identifying, refining, and prioritizing key issues in lymphoma care, ensuring that the final recommendations were informed by multidisciplinary expertise and based on clinical evidence. This study was conducted between June and December 2024, under the leadership of a scientific board composed of recognized lymphoma experts. The project was sponsored by the IRCCS Istituto Tumori ‘Giovanni Paolo II’ Bari (Italy) by a grant from the Italian Ministry of Health (Ricerca Corrente 2025, del. n. 197/2025), recognizing both the scientific relevance of the initiative and its significant social impact in addressing the individual needs of patients with lymphoma. The project involved the participation of four central-southern Italian regions – Abruzzo, Basilicata, Molise, and Puglia – with the aim of defining a shared diagnostic-therapeutic pathway to optimize the care of lymphoma patients. Following the completion of the initial NGT session, the Delphi questionnaire was developed and distributed to participating clinicians. The iterative nature of the Delphi survey allowed for the refinement of statements, ensuring that the final recommendations accurately reflected the collective expertise and consensus of the panel.

**Figure 1:**
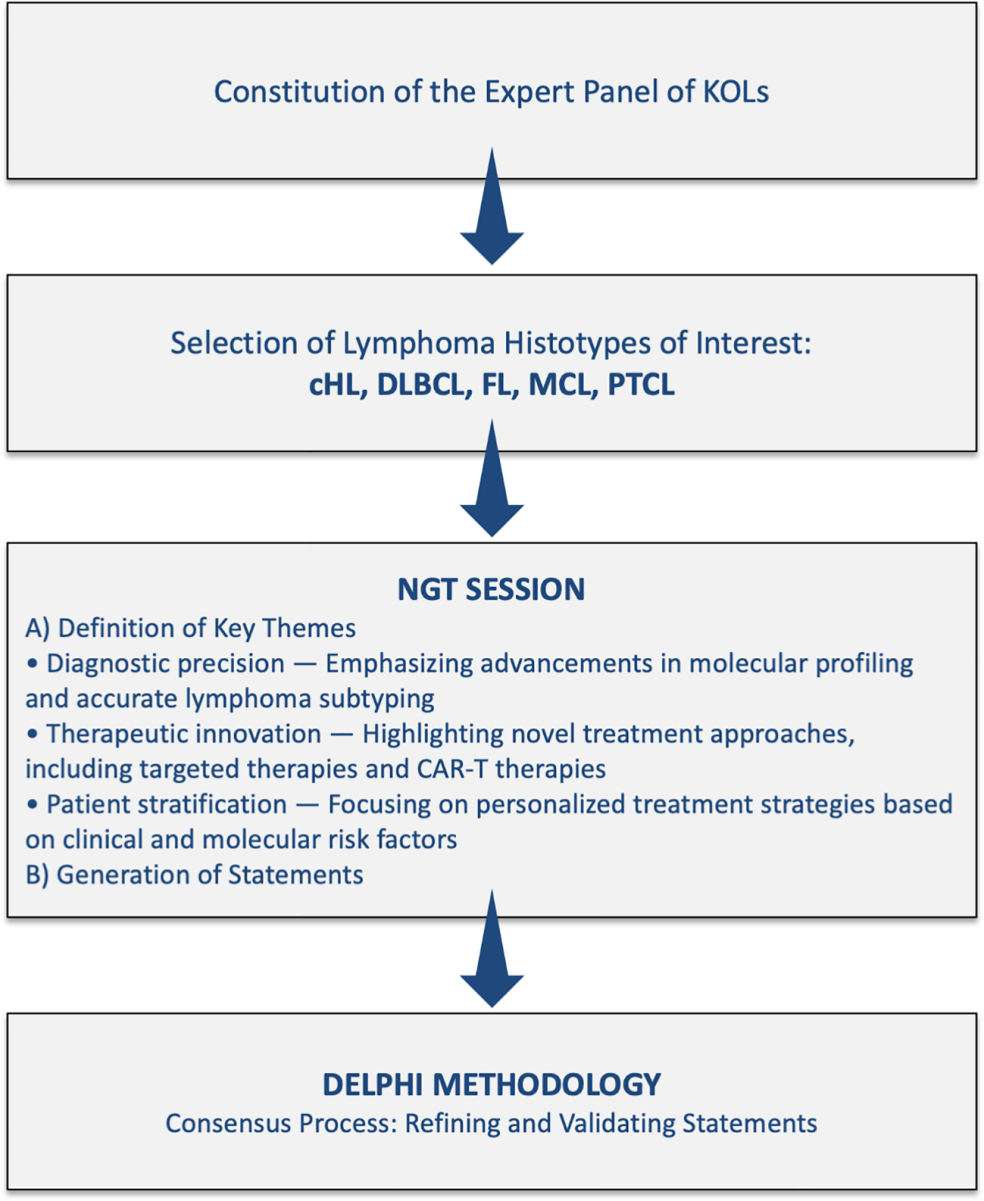
Study flow chart and consensus process for the project “Right Path 4 Lymphomas”.

**Figure 1** shows the Study flow chart and consensus process for the project “Right Path 4 Lymphomas”.

### 2.1 Participants

The expert panel consisted of key opinion leaders (KOLs) with extensive clinical experience in the diagnosis and treatment of lymphoma. The expert panel included a multidisciplinary group of clinicians with specialized expertise in the diagnosis and treatment of lymphoma: onco-hematologists with at least 10 years of experience in lymphoma diagnosis and treatment, and members of scientific societies in lymphoma and hematology (Italian Lymphoma Foundation/Fondazione Italiana Linfomi, FIL; Italian Hematology Society/Società Italiana di Ematologia, SIE); hemolympho-pathologists with expertise in the diagnosis and molecular characterization of lymphomas. This multidisciplinary composition ensured that a broad range of perspectives informed the consensus-building process, enhancing the applicability and relevance of the final recommendations.

The experts included in the panel were subsequently divided into five improvement groups, each dedicated to a specific lymphoma histotype.

### 2.2 Phase 1: NGT methodology

The first phase of the project involved the use of the NGT to generate and prioritize expert statements addressing critical diagnostic and therapeutic aspects of lymphoma management. The NGT process began with a structured in-person group discussion facilitated by a methodologist, who provided participants with a detailed explanation of the methodology before initiating the session. A professional medical writer was present to document the ideas generated by the panelists throughout the session.

During the meeting, the KOL panel decided to dedicate the discussion to five lymphoma histotypes that best represent the evolution of diagnostic and treatment approaches in recent years:

- Classical Hodgkin lymphoma (cHL);
- Diffuse large B-cell lymphoma (DLBCL);
- Follicular lymphoma (FL);
- Mantle cell lymphoma (MCL);
- Peripheral T-cell lymphomas (PTCLs), including breast implant-associated anaplastic large cell lymphoma (BIA-ALCL).

The project focused on the adult population.

The NGT session utilized verbal input from participants as a systematic method for capturing individual expert opinions and generating consensus, in accordance with the established NGT framework. All participants actively contributed to the discussions, ensuring equal engagement in the scientific debate and promoting the development of a robust list of key topics. Ideas proposed by the experts were documented on a flip chart and subsequently organized into a structured report summarizing the meeting’s findings (15, 16). The NGT method is a widely used, structured small-group discussion technique designed to achieve consensus by collecting individual responses to a series of questions posed by a moderator. It is followed by a group-wide process of idea sharing and prioritization. This approach prevents the domination of discussions by a single participant, encourages equal participation, and results in a ranked list of solutions or recommendations that reflect the group’s collective preferences (15, 16).

During this session, the expert panel generated a total of 270 statements, which were categorized into three key themes:

1. Diagnostic precision, emphasizing advancements in molecular profiling and accurate lymphoma subtyping;
2. Therapeutic innovation, highlighting novel treatment approaches, including targeted therapies and CAR-T therapies;
3. Patient stratification, focusing on personalized treatment strategies based on clinical and molecular risk factors.

The experts worked within the groups, and eight key questions common to all groups were presented (**Table 1**). They provided individual input and perspectives in response to these questions. These considerations were then analyzed and synthesized into statements. For each lymphoma subtype, a set of statements was developed for each key question.

- The cHL group generated 32 statements.
- The DLBCL group generated 49 statements.
- The FL group generated 52 statements.
- The MCL group generated 48 statements.
- The PTCL group generated 89 statements.

**Table 1:** Key questions summary.

Key question	Description
Key question 1	Which clinical signs and symptoms may suggest the presence of the disease?
Key question 2	Which diagnostic tests are recommended for achieving an accurate diagnosis?
Key question 3	Which medical specialists should be involved in the diagnostic process?
Key question 4	Which prognostic factors should be taken into account?
Key question 5	What is the optimal therapeutic approach according to the stage of the disease?
Key question 6	Which criteria should be applied to assess treatment response?
Key question 7	Which clinical visits and diagnostic tests are required for appropriate follow-up, and how frequently should they be conducted?
Key question 8	What salvage treatment options are recommended for relapsed or refractory disease?

The prioritized statements formed the basis for the Delphi process questionnaire, which was used in the second phase of the project. The complete set of statements produced by each group was incorporated into the Delphi questionnaire, which was distributed to all participating KOLs. Importantly, each KOL responded to the questionnaire independently, regardless of their original group assignment.

### 2.3 Phase 2: Delphi methodology

The Delphi methodology was employed in the second phase to achieve consensus among KOLs on diagnostic tools, treatment strategies, and follow-up practices in lymphoma care. Originally developed by the RAND Corporation, the Delphi method is a validated approach for consensus-building and group decision-making across disciplines (16–19). In clinical research, it is widely used to address complex challenges by synthesizing expert opinions into evidence-based recommendations (20). The primary objective of the Delphi process was to refine the prioritized statements generated during the NGT session and achieve consensus on the level of agreement or disagreement among the expert panel. The process involved a round of anonymous voting, during which participants evaluated each statement using a 5-point Likert scale (1 = complete disagreement, 5 = complete agreement). Then, the panel’s responses were analyzed, and controlled feedback was provided to refine statements and address areas of disagreement.

Consensus was defined based on a predefined agreement threshold (≥75%), calculated by summing the percentages of responses scoring 4 (agreement) and 5 (full agreement), as indicated by participants on a 5-point Likert scale ranging from 1 (complete disagreement) to 5 (complete agreement). The ≥75% threshold was predefined and reflects widely accepted methodological standards for Delphi processes, which commonly adopt cut-offs between 70% and 80% to define expert consensus without requiring unanimity. This threshold balances methodological rigor with feasibility in multidisciplinary panels and is supported by published guidelines and literature on structured consensus-building techniques (17).

By combining the structured statement generation of the NGT phase with iterative validation through the Delphi process, this methodology ensured that the project incorporated robust, multidisciplinary input and culminated in evidence-based, actionable recommendations for improving lymphoma care.

## 3 Results

Results from the “Right Path 4 Lymphomas” project offer a comprehensive analysis of key aspects of lymphoma management, demonstrating a high level of consensus on current diagnostic and therapeutic approaches. The expert panel achieved substantial agreement on most statements, with consensus reached on 264 out of 270 statements (97.8%), while only six statements (2.2%) fell below the predefined consensus threshold. Areas of disagreement were analyzed in detail to identify the underlying reasons and assess their implications for clinical practice. These findings highlight the value of a structured consensus-building methodology in incorporating multidisciplinary perspectives and generating evidence-based recommendations to support clinical decision-making.

### 3.1 General statements on diagnosis, management, and follow-up

This section presents consensus statements on the diagnosis, management, and follow-up of lymphoma.

Consensus was reached on the necessity of histopathological diagnosis, with excisional biopsy of the affected tissue (primarily lymph node or extranodal sites) as the preferred approach. Core biopsy and fine-needle aspiration biopsy were considered secondary options to be used when excisional biopsy was not feasible. Diagnosis should include immunohistochemistry (IHC) and molecular characterization of histological samples, following the 2022 WHO and 2022 ICC classifications for lymphoid malignancies (6, 7).

The importance of a multidisciplinary approach was strongly endorsed by the panel, emphasizing the key roles of surgeons, radiologists, nuclear medicine specialists, molecular biologists, pathologists, and onco-hematologists in achieving an accurate diagnosis and staging based on the Lugano criteria (21). Additionally, a cardio-oncologist may be required for baseline cardiovascular assessment, risk stratification, and monitoring. Multidisciplinary evaluation was also recommended before the initiation of antineoplastic therapy, particularly for fertility counseling and preservation in young patients. The panel highlighted the value of multidisciplinary tumor boards for discussing complex cases, ideally within teams specialized in lymphoid malignancies.

The panel further emphasized the importance of vaccination assessment before initiating oncologic treatment, which aligns with recent FIL recommendations for lymphoma patients (22).

For disease staging and treatment response assessment, CT and PET/CT scans are recommended according to the Lugano criteria at staging, interim evaluation, and at the end-of-treatment assessment for all histotypes covered in this consensus. The DS should be used to evaluate treatment response. In cases where bone marrow biopsy was positive at diagnosis, a repeat biopsy after induction therapy is recommended. Additional diagnostic evaluations should be tailored based on the site of involvement, such as peripheral blood flow cytometry for leukemic disease or endoscopic assessment for gastrointestinal involvement. Blood tests at staging should include hepatitis B virus (HBV), hepatitis C virus (HCV), and Human immunodeficiency virus (HIV) screening, as well as hemolysis tests when clinically indicated.

Prognostic scores and risk factors were discussed for each histotype and are presented in dedicated sections. These prognostic models were indicated as central to guide therapeutic decisions.

Beyond treatment strategies, the panel emphasized the importance of supportive care measures. Antimicrobial and antiviral prophylaxis should be considered during induction therapy, based on patient age and treatment regimen (22). HBV prophylaxis was recommended for patients with occult HBV infection.

The need for long-term monitoring of treatment-related toxicities was widely recognized, particularly for cardiac, pulmonary, and secondary malignancies, depending on the type of chemotherapy or radiotherapy (RT) received, individual and familial risk factors, and age at treatment and at follow-up evaluation (23). However, consensus was not unanimous on whether patients should continue follow-up at specialist centers beyond 5 years after achieving disease remission.

Lifestyle modifications, including regular physical activity and adherence to a Mediterranean diet, have been shown to reduce cardiovascular risk and improve quality of life in lymphoma survivors (24). The panel strongly supported incorporating these strategies into survivorship care. Furthermore, agreement was reached on age- and sex-appropriate oncologic screenings as part of routine follow-up for lymphoma survivors (25).

Routine imaging and blood tests for relapse detection were broadly agreed until the first 24 months following the completion of therapy. However, PET/CT scans were not recommended for routine follow-up, except in specific high-risk scenarios. These scenarios primarily involve early relapse detection within 12 months post-induction therapy in DLBCL and progressive disease monitoring in high-risk MCL patients on ibrutinib, to facilitate timely referral for CAR-T therapy.

### 3.2 Hodgkin lymphoma

Results from the Delphi methodology on classical Hodgkin lymphoma (HL) demonstrated strong consensus regarding prognostic tools and therapeutic approaches. These findings provide a valuable framework for establishing shared diagnostic-therapeutic pathways aimed at optimizing patient outcomes in HL (**Supplementary Table 1**).

The histopathological diagnosis of HL must follow the criteria outlined in the 5th edition of the WHO classification (26). There are four recognized subtypes of HL: nodular sclerosis, mixed cellularity, lymphocyte-rich, and lymphocyte-depleted. The typical immunophenotype of HL is CD15+, CD30+, PAX-5+ (weak), CD3-, CD20- (majority), CD45-, CD79a-. Epstein-Barr encoding region *in situ* hybridization is recommended at initial diagnosis (cHL: EBER+/-), with additional markers (e.g., MUM-1, BOB-1, OCT-2) considered in selected cases. Nodular lymphocyte-predominant HL remains a distinct pathological, biological, and clinical entity and was not addressed by the panel.

Beyond the European Organisation for Research and Treatment of Cancer, German Hodgkin Study Group and National Comprehensive Cancer Network classifications for favorable/unfavorable early-stage disease (stage I–II) and the International Prognostic Score (IPS) for advanced-stage disease (stage III–IV), the panel evaluated the prognostic role of interim PET (PET2) with currently available induction therapies in Italy. However, no consensus was reached on the role of PET2 in brentuximab vedotin-doxorubicin, vinblastine, and dacarbazine (BV-AVD) and the need for treatment intensification in PET2-positive (DS 4–5) cases. The necessity of repeat biopsy in the event of positive PET2 was debated, indicating some divergence in clinical practice regarding its management (27).

For favorable early-stage disease, treatment typically consists of a short-course ABVD (doxorubicin, bleomycin, vinblastine and dacarbazine) regimen followed by involved-site RT (IS-RT). Unfavorable early-stage disease is treated with ABVD followed by IS-RT or two cycles of ABVD followed by four cycles of AVD, as per the RATHL trial (28). Since novel agents are currently not approved in Italy for early-stage disease, participation in clinical trials is encouraged. For stage III disease, treatment options include full-course ABVD or RATHL-based strategies in PET2-negative patients. In stage IV disease, the incorporation of BV-AVD is the gold-standard first-line therapy for eligible patients. Nivolumab-AVD represents a promising alternative with better tolerability, but it has not yet been approved in Italy (29). For patients with a negative end-of-treatment (EOT) PET, RT consolidation is no longer required. Escalated BEACOPP (bleomycin sulfate, etoposide phosphate, doxorubicin hydrochloride, cyclophosphamide, vincristine sulfate, procarbazine hydrochloride, and prednisone), while less frequently used as first-line therapy, remains an option for PET2-positive (DS 4) ABVD-treated patients who are eligible for intensification. The BrECADD (brentuximab vedotin, etoposide, cyclophosphamide, doxorubicin, dacarbazine, and dexamethasone) regimen is not yet approved in Italy for either front-line therapy or treatment intensification (30). Recently, the sequential use of BV and AVD followed by BV has become available for older patients (>60 years) with stage IV disease who are ineligible for bleomycin (31).

For refractory cHL (ABVD PET2 DS 5, EOT PET DS 4–5, or relapse within 3 months), as well as relapsed disease, autologous stem cell transplant (ASCT) remains the cornerstone of therapy for young and eligible patients. Preferred salvage regimens include BEGEV (bendamustine, gemcitabine, vinorelbine), ICE (ifosfamide, carboplatin and etoposide), or DHAP (dexamethasone, high-dose cytarabine and cisplatin). Immune checkpoint inhibitors (CPIs), such as pembrolizumab and BV, play a critical role in bridging patients to ASCT, with a preference for pembrolizumab based on the KEYNOTE-204 trial (32).

Currently, no combinations of BV or CPIs with chemotherapy are approved as salvage therapy prior to ASCT in Italy, and participation in clinical trials is strongly encouraged. The use of BV as post-transplant consolidation has shown to improve progression-free survival (PFS) in high-risk patients (33).

For post-ASCT relapse, treatment is primarily based on CPI therapy, with allogeneic stem cell transplantation (allo-SCT) to be considered for eligible patients with an available donor. In older or non-ASCT-eligible patients with relapsed/refractory (R/R) disease, treatment consists of CPI or BV, while awaiting more mature data and new drug combinations.

### 3.3 Diffuse large B-cell lymphoma

Results from the Delphi round on diffuse large B-cell lymphoma (DLBCL) provide a comprehensive framework for its diagnosis and treatment, emphasizing the importance of a multidisciplinary approach alongside advancements in diagnostic technologies and therapeutic strategies (**Supplementary Table 2**).

DLBCL is the most prevalent subtype of B- NHL, accounting for approximately 30–40% of cases in high-income countries. Its diagnosis should follow 2022 WHO and 2022 ICC recommendations (6, 7), requiring immunohistochemical and molecular characterization of histological samples, including markers such as CD45, CD20, CD19, CD79a, PAX5, CD3, CD5, BCL6, CD10, BCL2, c-MYC, Ki67, and IRF4/MUM1. Accurate classification of DLBCL by cell of origin is crucial, as it directly impacts treatment selection and prognosis. DLBCL can be categorized into three primary subtypes: germinal center B-cell-like (GCB-like), activated B-cell-like, and unclassified. Gene expression profiling is a valuable tool for identifying distinct molecular signatures associated with prognosis. However, in routine clinical practice, the Hans classifier, based on CD10, BCL6, and IRF4/MUM1 expression, serves as the most practical surrogate.

In cases where high-grade cytology, high MYC (>40%) and BCL2 (>50%) expression, and the GCB phenotype are present, FISH analysis is recommended to assess for MYC and BCL2 rearrangements, which define high-grade B-cell lymphoma with MYC/BCL2 rearrangements, according to the 2022 WHO classification. Particularly, double-hit DLBCL with MYC/BCL2 rearrangements is poorly responsive to standard immunochemotherapy and may represent a distinct clinical entity, based on recent gene expression studies (34). Ongoing research continues to refine the molecular taxonomy of DLBCL, identifying novel genetic subsets with varying responses to frontline therapy (35). While the LymphGen algorithm has been proposed to enhance DLBCL classification, it is not yet recommended for routine clinical use, as nearly 40% of cases remain unclassified.

Beyond standard staging procedures, additional investigations may be required to assess extranodal disease involvement, such as endoscopy in cases of suspected gastrointestinal involvement or a central nervous system (CNS)-directed work-up when neurological symptoms are present. There is a general consensus that approximately two-thirds of DLBCL patients achieve long-term remission with first-line therapy, and those who remain relapse-free for 24 months exhibit a life expectancy similar to the general population (36). However, accurate baseline risk stratification is critical for optimizing treatment. In addition to molecular and pathological factors, clinical prognostic models remain essential, including the International Prognostic Index (IPI), the age-adjusted IPI for patients under 60 years old, and the Revised IPI, which aid in determining treatment intensity and supportive care needs. Recognizing that DLBCL primarily affects older adults, the panel strongly recommended a baseline geriatric assessment to guide treatment decisions. Tools such as the Comprehensive Geriatric Assessment (CGA), which evaluate functional status, comorbidities, cognitive function, social support, and nutrition, help identify vulnerabilities that may impact treatment tolerance and outcomes (37).

The standard first-line treatment has long been R-CHOP (rituximab, cyclophosphamide, doxorubicin, vincristine, and prednisone). Recent evidence supports the polatuzumab vedotin plus rituximab–cyclophosphamide–doxorubicin–prednisone (R-CHP) regimen as a superior alternative in terms of reduced risk of disease progression, relapse and death to R-CHOP in advanced stage intermediate- to high-risk disease, with a specific benefit observed in non-GCB subtypes (38). The use of liposomal anthracycline is suggested for patients with cardiovascular comorbidities. The need for CNS prophylaxis should be assessed on a case-by-case basis, with the CNS-IPI serving as the primary tool for risk stratification. In high-risk patients, such as those with testicular or renal involvement, CNS prophylaxis with high-dose methotrexate is recommended. However, the benefits of CNS prophylaxis should be tailored to each patient’s clinical context. In frail older patients, R-mini-CHOP is the preferred regimen. For patients with double-hit or triple-hit lymphomas, the dose-adjusted EPOCH-R (etoposide, prednisone, vincristine, cyclophosphamide, doxorubicin, and rituximab) regimen should be used when eligibility criteria are met (39).

Treatment response should be assessed using EOT PET/CT scans, with response quantified using the DS. In the R/R setting, treatment strategies depend on the timing of relapse and transplant eligibility. For patients experiencing early relapse within 12 months, CAR-T therapy (axicabtagene ciloleucel, lisocabtagene maraleucel) is the preferred option, with a strong emphasis on the timely collection of lymphocytes to ensure eligibility (40). In cases of late relapse, salvage chemotherapy followed by ASCT remains the standard of care for eligible patients. For transplant-ineligible patients, a chemo-free approach with tafasitamab-lenalidomide is preferred, while polatuzumab–rituximab–bendamustine remains a viable alternative (41, 42). In the third-line setting, CAR-T therapy (axicabtagene ciloleucel, lisocabtagene maraleucel, tisagenlecleucel) and bispecific antibodies, such as glofitamab and epcoritamab, have durable remission and prolonged survival (43–46). Loncastuximab tesirine represents a promising option with valid responses and an acceptable safety profile (47). The role of allogeneic transplantation in post-CAR-T relapsed patients remains under discussion, particularly given the availability of bispecific antibodies. These findings highlight the increasing importance of immunotherapies in reshaping the therapeutic landscape of DLBCL.

For patients with primary CNS lymphoma, the rituximab, high-dose methotrexate, cytarabine, and thiotepa (R-MATRIX) regimen followed by autologous hematopoietic stem cell transplantation (HSCT) remains the standard of care (48). In older or unfit patients, rituximab, methotrexate, procarbazine, and vincristine (R-MPV) is the preferred regimen (49). Salvage therapy for relapsed or refractory primary CNS lymphoma includes RT, temozolomide, and lenalidomide, but no universally accepted gold-standard regimen has been established. In primary mediastinal large B-cell lymphoma, recent phase III data suggest that mediastinal RT may be omitted after rituximab-chemotherapy induction, provided that the EOT PET/CT scan (DS 1–3) is negative (50).

The panel also addressed special populations within the DLBCL setting. In HIV-positive patients with well-controlled viral loads on antiretroviral therapy, standard DLBCL treatment regimens should be administered (51). For post-transplant lymphoproliferative disorders, initial management should involve immunosuppression reduction, with rituximab monotherapy as the first-line treatment and rituximab-chemotherapy reserved for selected cases (52).

### 3.4 Follicular lymphoma

Results from the Delphi consensus on follicular lymphoma (FL) provide critical insights into its diagnostic and therapeutic management (**Supplementary Table 3**). These findings emphasize the need for a comprehensive, evidence-based, patient-centered approach to managing this indolent lymphoma.

The diagnosis of FL requires an accurate assessment according to the 2022 WHO and 2022 ICC classifications for lymphoid malignancies (6, 7). These include FL grades 1, 2, and 3A, as well as classic FL. Immunohistochemical profiling typically demonstrates CD20+, CD10+, BCL2+, CD23+/-, CD5-, BCL6+ and/or LMO2+. However, FL may occasionally present as CD10- or BCL2-. In 85–90% of cases, a BCL2/IGH (t14;18)(q32; q21) rearrangement is detected by FISH, leading to BCL2 overexpression, which strongly supports the diagnosis (53). This rearrangement should be investigated in FL cases lacking BCL2 expression on IHC to distinguish FL from other low-grade B-cell lymphomas, such as marginal zone lymphoma. FISH analysis for *BCL6* gene translocation is also recommended, as it supports a diagnosis of FL.

Certain FL subtypes exhibit distinct biological features (54). FLs arising in inguinal sites more frequently show diffuse growth patterns, 1p36 deletion, absence of BCL2 rearrangement, and CD23 positivity. In high-grade FLs that lack CD10 expression and BCL2 rearrangement, immunohistochemical detection of *IRF4*/*MUM1* is recommended, as high expression of this marker correlates with *IRF4* (*MUM1*) gene rearrangement, leading to a diagnosis of *IRF4* (*MUM1*)-rearranged large B-cell lymphoma. High-grade FL 3B (7) and follicular large cell lymphoma (2022 WHO classification) are closely related entities and are managed similarly to DLBCL (6, 7). In these cases, MYC expression should be assessed. If MYC protein levels exceed 40%, further investigation of *MYC* gene rearrangement is recommended to rule out transformation into high-grade lymphoma with BCL2 and MYC double rearrangement. Duodenal-type FL is recognized as a distinct entity, with most patients presenting with localized, clinically indolent disease. While its morphology, immunophenotype, and genetic profile resemble those of nodal FL grade 1–2, its clinical course remains unique.

The diagnostic work-up for FL follows standard protocols used for other lymphoma subtypes but may also include peripheral blood flow cytometry to detect leukemic involvement. Beyond standard prognostic models, such as Follicular Lymphoma International Prognostic Index (FLIPI) and FLIPI-2, additional tools such as the DS at EOT assessment and disease progression within 24 months have been recognized for their prognostic significance (55). Emerging biomarkers, such as total metabolic tumor volume, require further validation and standardization before routine clinical adoption (56). The use of minimal residual disease (MRD) monitoring by reverse transcription PCR remains a topic of debate. While its prognostic value is acknowledged, MRD is not yet a standard tool in clinical practice (57). Multiparametric flow cytometry, being more accessible, could serve as a practical alternative. Additionally, molecularly guided prognostic models, such as m7-FLIPI, hold promise but are not yet widely implemented.

Therapeutic strategies for FL were discussed according to disease presentation. In localized disease (stage I–II with contiguous nodal involvement), strong consensus was reached on IS-RT (24 Gy), as the preferred approach (58), while anti-CD20 monoclonal antibody monotherapy (rituximab) was considered in cases where RT is contraindicated (59). In advanced-stage, asymptomatic, and low-tumor-burden disease, a watch-and-wait strategy was strongly endorsed. The use of rituximab monotherapy for patients with advanced-stage, low-tumor-burden FL was discussed and considered an option for selected cases (60).

For advanced-stage FL meeting the Groupe d’Etude des Lymphomes Folliculaires (GELF) criteria, chemoimmunotherapy was favored, with rituximab-bendamustine preferred over rituximab-CHOP as the first-line option (61). In patients with intermediate-to-high FLIPI scores, rituximab may be replaced by obinutuzumab in combination with bendamustine or CHOP (62). When histologic transformation is suspected but cannot be confirmed by biopsy, rituximab- or obinutuzumab-CHOP was preferred (53). Patients with a history of cardiac disease should receive liposomal doxorubicin instead of standard doxorubicin. For those achieving a complete or partial metabolic response at the EOT PET/CT scan, maintenance therapy with the anti-CD20 monoclonal antibody used in induction (rituximab or obinutuzumab) was recommended every 8 weeks for 12 cycles. In frail patients or those over 80 years old, treatment should be individualized using reduced-toxicity regimens, such as rituximab monotherapy, reduced-dose bendamustine, or R-CVP. The panel also agreed on the necessity of HCV eradication either before initiating FL treatment or at the end of induction therapy, depending on disease burden and the urgency of oncologic intervention.

In relapsed disease, many patients benefit from an initial period of observation. Disease recurrence should be histologically confirmed, particularly when associated with elevated lactate dehydrogenase (LDH), non-homogeneous adenopathy growth, extranodal involvement, bulky disease (>7 cm), or systemic symptoms (53). Areas of high SUVmax on PET/CT scan (especially >13) raise suspicion of histologic transformation and should be biopsied (53). The GELF criteria should continue to guide treatment initiation in relapsed FL, as they do in newly diagnosed cases. Enrollment in clinical trials should be considered whenever possible.

For patients experiencing first relapse within 24 months, particularly those with bulky disease or high SUVmax, salvage chemotherapy followed by ASCT should be considered but the use of this approach is progressively declining in light of the presence of effective third-line options. In patients with late relapse (beyond 24 months) or those ineligible for transplant, a chemo-free approach with rituximab-lenalidomide (R2 regimen) was preferred (63). The combination of tafasitamab-lenalidomide-rituximab is a promising option but has not yet been approved in Italy (64). For patients in a second relapse, treatment with mosunetuzumab or CAR-T therapy (axi-cel, tisa-cel) represent the best options. Fixed-duration mosunetuzumab therapy has demonstrated durable benefits in this patient setting, with a manageable safety profile (65). Axicabtagene ciloleucel and tisagenlecleucel showed high rates of durable responses (66, 67).The choice between bispecific antibodies and CAR-T therapy remains under discussion.

### 3.5 Mantle cell lymphoma

Results from the Delphi consensus on mantle cell lymphoma (MCL) provide a comprehensive overview of its diagnostic and therapeutic management (**Supplementary Table 4**). The findings underscore the complexity of MCL, emphasizing the need for precise diagnostics, individualized treatment strategies, and multidisciplinary care.

MCL accounts for approximately 5–7% of all lymphomas and presents with a heterogeneous clinical course (68). While some cases exhibit an indolent, leukemic non-nodal phenotype (10–15% of cases) that may remain asymptomatic for years, the majority display aggressive behavior, necessitating immediate treatment (69). Diagnosis relies on histological examination of nodal or extranodal biopsies, as MCL frequently involves the gastrointestinal tract, bone marrow, and peripheral blood (70). The immunophenotypic profile typically includes CD20+, CD79a+, CD19+, CD5+, cyclin D1+, IgM+, IgD+, SOX11+, CD43-, lambda chain+, kappa chain-/+, CD10-, CD23-, and BCL6-. Genetically, MCL is characterized by the t (11, 14)(p13;q32) translocation, which drives cyclin D1 overexpression, a defining diagnostic feature present in over 95% of cases (6, 7, 71). The overexpression of SOX11, detected in more than 90% of cases, further supports the diagnosis. However, SOX11-negative variants tend to follow a more indolent course, with a higher prevalence of leukemic non-nodal involvement and a lower risk of disease progression (68, 71). Indeed, the 2022 WHO and ICC classifications recognize three MCL subtypes: classic nodal/extranodal MCL, non-nodal leukemic MCL, and *in situ* mantle cell neoplasia (6, 7). From a cytological perspective, four morphological variants are defined: blastoid, pleomorphic, small-cell, and marginal zone-like, with blastoid morphology correlating with a poorer prognosis (6, 7).

Advances in NGS have identified recurrent gene mutations involved in cell cycle regulation and stress responses, although *TP53* mutations remain the most clinically relevant. The presence of *TP53* mutations, independent of other factors, is associated with chemotherapy resistance and inferior survival, making *TP53* status evaluation essential before initiating treatment. Given the diagnostic complexity, the panel emphasized the crucial role of pathologists in integrating morphological, immunophenotypic, and molecular data to ensure accurate classification and risk stratification.

Several key prognostic markers and models were highlighted for their role in patients’ stratification. The Mantle Cell Lymphoma IPI (MIPI) incorporates clinical variables such as age, Eastern Cooperative Oncology Group performance status, LDH, and leukocyte count, while the c-MIPI score integrates Ki-67 proliferative index, with a Ki-67 >30% recognized as a marker of aggressive disease. *TP53* mutation status, assessed through NGS, is a strong predictor of poor response to conventional chemotherapy across all age groups, reinforcing the need for targeted therapeutic strategies. Although the IGHV (immunoglobulin heavy chain variable region) mutational status may also provide prognostic insight, it did not achieve consensus due to limited applicability in routine practice.

The panel emphasized the importance of tailoring treatment based on age, fitness, and prognostic factors, while also underscoring the need for comprehensive supportive care. In younger patients with advanced-stage MCL, the standard approach consists of R-CHOP alternated to intensive R-DHAP immunochemotherapy, with ASCT as consolidation therapy and rituximab maintenance. Recent data from the TRIANGLE trial have demonstrated the potential benefit of incorporating ibrutinib into induction and maintenance therapy, which could overcome negative prognostic factors and potentially replace ASCT (72). This regimen has recently been approved in Italy due its high innovative value. In patients over 65 or those ineligible for ASCT, bendamustine-aracytin could be preferred for fit patients (73). The combination of acalabrutinib with bendamustine-rituximab followed by acalabrutinib until progression and rituximab maintenance has recently become available in Italy following the publication of the ECHO trial (74). The potential role of chemo-free options in frontline therapy for older patients, particularly those with TP53 mutations, has been recognized, as it improves PFS without affecting OS. However, regulatory approval is still pending (53).

For relapsed MCL, BTKi are considered the first option, regardless of early or late relapse, with ibrutinib currently the only approved BTKi in Italy. The panel recommended repeating a biopsy at relapse whenever feasible and re-evaluating TP53 mutation status to inform treatment decisions. Patients receiving ibrutinib require close monitoring, as rapid progression necessitates early referral to CAR-T therapy (brexucabtagene autoleucel), which remains the preferred approach for eligible patients (74, 75). In Italy, CAR-T therapy in partial response is available through clinical trials. The panel stressed the need for vigilant monitoring in high-risk patients on ibrutinib and early consultation with CAR-T centers. For patients who are ineligible for CAR-T therapy, the non-covalent BTKi pirtobrutinib is the preferred option (76). Venetoclax, either as monotherapy or combined with BTKi, could be effective in high-risk patients, although it remains off-label in this setting (77).

Bispecific antibodies have demonstrated promising results, highlighting a shift toward precision medicine in MCL (78). Allo-SCT remains an option for young, fit patients who relapse after CAR-T therapy, although its role continues to evolve in the context of novel immunotherapies (79).

### 3.6 Peripheral T-cell lymphomas and breast implant-associated anaplastic large cell lymphoma

Results from the Delphi consensus on PTCLs provide a comprehensive and nuanced understanding of the diagnostic and therapeutic paradigms for these heterogeneous and challenging malignancies (**Supplementary Table 5**). The findings emphasize the importance of precise diagnostics, risk-adapted therapy, and multidisciplinary collaboration. The panel focused specifically on PTCLs, which constitute the most common T-cell NHLs (T-NHLs), as well as BIA-ALCL.

The histologic diagnosis of PTCLs should be performed by an expert hemolympho-pathologist following the 2022 WHO and 2022 ICC classifications for lymphoid malignancies (6, 7). PTCLs encompass several subtypes, including PTCL not otherwise specified (PTCL-NOS), ALK-positive ALCL, ALK-negative ALCL, angioimmunoblastic-type T-cell lymphoma (AITL/T follicular helper [TFH] lymphoma), and TFH lymphoma NOS (2022 WHO classification). Immunohistochemical evaluation should include CD20, CD3, CD10, BCL6, Ki-67, CD5, CD30, CD2, CD4, CD8, CD7, CD56, CD21, CD23, PD1/CD279, TCRβ, TCRδ, TIA-1, granzyme B, and perforin (80). To further classify PTCL subtypes, TFH-associated markers (CD10, BCL6, PD1/CD279, ICOS, CXCL13) and cytotoxic T-cell markers (TIA-1, granzyme B, perforin) should also be evaluated. T-cell receptor (TCR) gene rearrangements should be assessed, while FISH for DUSP22 and TP63 rearrangements is recommended in *ALK*-negative ALCL. Furthermore, Epstein-Barr encoding region *in situ* hybridization is a mandatory test for identifying Epstein-Barr virus-related T-cell lymphomas. Additional analyses, including flow cytometry on peripheral blood and aspirated samples for clonality assessment, may be required in selected cases. These diagnostic strategies align with recent updates on T-NHLs, emphasizing the role of histopathology, immunophenotyping, and molecular markers in accurate subtyping and prognostication (81).

Prognostic assessment should incorporate the IPI, which has been adapted for PTCLs, alongside the prognostic index for PTCL-NOS (PIT) and its modified version including Ki-67 expression. The prognostic significance of DUSP22 positivity in ALK-negative ALCL remains under evaluation in the context of current frontline therapies (82). Additionally, the recently developed Prognostic Index for Relapsed T-cell Lymphomas (PIRT) score exemplifies advancements in risk stratification for relapsed and refractory PTCLs.

Several areas of debate highlight the evolving landscape of PTCL management. Treatment strategies are stratified by subtype, patient age, and disease stage. For PTCL-NOS, AITL, and TFH lymphomas, induction therapy with cyclophosphamide, vincristine, doxorubicin, etoposide, and prednisone (CHOEP), CHOP, or CHOP-like regimens is preferred (83). For eligible patients achieving remission, the panel strongly recommended ASCT as frontline consolidation. In ALK-positive and ALK-negative ALCL, BV-CHP is the recommended first-line therapy, while ASCT consolidation should be considered for ALK-negative ALCL and high-risk ALK-positive cases, particularly those with high-risk IPI, extranodal involvement, or residual disease (84). RT consolidation may be considered in rare cases of early-stage disease or transplant-ineligible patients.

For relapsed or refractory PTCLs, salvage regimens, such as ICE or DHAP, followed by ASCT, are endorsed for transplant-eligible patients. In subsequent treatment lines, novel targeted agents should be incorporated, such as BV for CD30-positive cases or ALK inhibitors for ALK-positive disease. Given the limited therapeutic options in this setting, participation in clinical trials is strongly encouraged to improve access to emerging therapies. Recent global evidence suggests that treatment outcomes in relapsed PTCLs can be significantly influenced by access to novel therapies, further underscoring the importance of integrating small-molecule inhibitors and targeted agents into the treatment paradigm (80). In young patients who relapse after ASCT, bridging therapy with novel agents followed by allo-SCT should be considered.

BIA-ALCL is a rare subtype of PTCL that arises in women with a history of textured breast implants and typically presents with effusion and breast swelling. Diagnosis is based on cytologic examination of the effusion fluid and multiple biopsies, with breast MRI aiding in both biopsy guidance and surgical planning. Tumor cells exhibit large anaplastic morphology, are positive for CD30 and variably for pan–T-cell markers, but are negative for ALK expression (85). Establishing a definitive diagnosis requires correlation with the patient’s history of breast implants. PET-positive lymph nodes should undergo biopsy to ensure accurate staging. Given the diagnostic complexity, expert pathologists should evaluate cases, and new diagnoses should be registered with the Italian Ministry of Health, which maintains a list of accredited specialists. A multidisciplinary team, including a plastic surgeon, is essential for comprehensive management.

Treatment of BIA-ALCL should be tailored to the disease stage. Most cases exhibit an indolent course with localized progression, for which complete surgical excision is strongly recommended. This involves removal of the implant, capsule, and any associated mass, considering contralateral implant removal, especially if textured (85). In cases of incomplete excision, the need for RT, additional surgery, or chemotherapy should be discussed within the multidisciplinary team. In rare cases of advanced disease (stages II–IV) with regional lymph nodes or distant organ involvement, systemic BV-CHP chemotherapy is recommended as the preferred frontline therapy.

## 4 Discussion

The “Right Path 4 Lymphomas” project is the first initiative involving multiple hematology centers and a multidisciplinary team of experts, aimed at developing consensus-driven strategies for lymphoma diagnosis and treatment. The project highlights the value of multidisciplinary collaboration, integrating the expertise of onco-hematologists, pathologists, radiologists, and subspecialists to promote standardized and comprehensive patient care across various lymphoma subtypes. The study findings reflect the complexity and ongoing evolution of diagnostic and therapeutic approaches, providing a structured framework to support clinical practice.

Statements developed through the NGT methodology were subsequently voted on during a Delphi process, achieving a high level of consensus. This approach underscores the importance of structured collaboration in formulating evidence-based recommendations for lymphoma management. However, areas of disagreement identified during the Delphi process highlight persistent challenges, emphasizing the need for further research to refine clinical strategies and address unresolved questions. In particular, the lack of consensus on MRD monitoring, the role of novel prognostic models, and the optimal sequencing of immunotherapies remain areas requiring further investigation.

Among the diagnostic advancements discussed, the comparative effectiveness of CAR-T therapies versus bispecific antibodies, especially in R/R FL, remains a topic of debate, with low agreement among panelists.

A key aspect of the discussions focused on the clinical application of MRD as a prognostic tool. The panel examined its potential to refine treatment decisions, particularly in advanced-stage HL, DLBCL, and MCL. The identification of high-risk patients was recognized as a critical factor, facilitated by molecular investigations and prognostic scoring systems that help guide treatment choices.

Consensus was also reached on first- and second-line treatment approaches, emphasizing the integration of innovative therapies tailored to disease stage and patient characteristics.

Despite these uncertainties, the project established a broad consensus on the diagnostic phase and classification of lymphomas, emphasizing the critical role of expert pathologists and the application of molecular biology techniques to ensure diagnostic accuracy. This consensus provides a strong foundation for tailored treatment decisions. Furthermore, unanimous agreement was reached on staging procedures and disease reassessment, particularly regarding follow-up imaging for high-risk patients who may benefit from innovative treatments, such as CAR-T therapy for refractory DLBCL and MCL.

The panel addressed emerging therapeutic advancements, particularly the anticipated approval of new drug combinations for high-risk patients in Italy. These include BV in the BrECADD regimen for high-risk HL and the potential extension of BV-AVD to stage III patients. The role of novel therapies, such as BiTEs and CAR-T therapy for high-risk DLBCL and high-grade lymphomas, as well as BTKi as a first-line option in high-risk MCL and older patients, was also discussed.

The integration of new therapies into second-line or salvage regimens was another key focus. The panel explored the use of BV or CPIs in HL salvage therapy, BiTE-based regimens in second-line DLBCL, and the potential application of tafasitamab-lenalidomide in second-line FL. Some areas of debate emerged, particularly regarding the role of ASCT in second-line FL and the need for clearer criteria to distinguish between patients eligible for CAR-T therapy versus BiTEs in third-line FL and DLBCL.

Overall, the “Right Path 4 Lymphomas” project provided a collaborative platform for experts to discuss key aspects of lymphoma diagnosis, treatment, and follow-up, in alignment with current therapeutic approvals in Italy. The consensus findings are consistent with international and European guidelines, including those from the European Society for Medical Oncology [(https://www.esmo.org/guidelines)] and the National Comprehensive Cancer Network [(https://www.nccn.org/guidelines/category_1)], while also taking into account Italian regulatory considerations.

The project resulted in the development of a consensus manuscript, offering clinicians practical, evidence-based guidance for lymphoma management. The document serves as a resource for decision-making in clinical practice, particularly in optimizing treatment strategies and standardizing patient care pathways. Additionally, it provides a structured framework for policymakers involved in approving and implementing new organizational or pharmacological approaches. The use of NGT and Delphi methodologies facilitated expert alignment, reinforcing their role in reaching consensus on complex clinical issues.

Given the continuous advancements in lymphoma diagnostics and therapeutics, periodic updates to the consensus document will be necessary to maintain its relevance. Future iterations may also extend discussions to less common lymphoma subtypes, ensuring the document remains applicable across a broad range of clinical scenarios. An acknowledged limitation of the present consensus is the absence of direct patient or caregiver involvement. Future updates should aim to integrate patient perspectives, particularly regarding quality-of-life considerations and long-term treatment preferences.

## 5 Conclusion

The results of the NGT and Delphi methodology provide a comprehensive framework for lymphoma diagnosis and treatment, reinforcing established clinical practices while highlighting key areas for innovation and further investigation. The high level of consensus achieved underscores the effectiveness of a structured methodology in generating robust, evidence-based recommendations. These findings serve as a foundation for developing standardized DTPs that optimize patient outcomes and ensure alignment with evolving standards of care in Italy.

## Data Availability

The original contributions presented in the study are included in the article/**Supplementary Material**. Further inquiries can be directed to the corresponding author.
