# Supplementary material for: Diagnostic and therapeutic pathways for lymphoma patients: expert consensus through Nominal Group Technique and Delphi methodology
Source: Front Oncol. 2025 Aug 13;15:1627175. doi: 10.3389/fonc.2025.1627175 (PMC12380629; doi:10.3389/fonc.2025.1627175)
Supplement: Supplementary file 1 [file DataSheet1.pdf]

Supplementary material

Supplementary Table 1. Results from the Delphi process on the clinical management of classical Hodgkin lymphoma (cHL).

**Key question 1: Which clinical signs and symptoms may suggest the presence of the disease?**		
Statements	***Consensus (%)***	***Decision***
B symptoms, which include fever, profuse night sweats, and significant weight loss (more than 10% of body weight within 6 months), are important indicators in the diagnostic suspicion of HL.	5: 68%<br>4: 32%<br>3: 0%<br>2: 0%<br>1: 0%	Consensus reached
In the presence of lymphadenopathy, it is important to assess whether the patient exhibits associated B symptoms (fever, night sweats, or weight loss), which may raise suspicion of lymphoma.	5: 53%<br>4: 26%<br>3: 21%<br>2: 0%<br>1: 0%	Consensus reached
Pruritus should not be classified as a B symptom. Although it is a common manifestation in patients with lymphoma, it is less specific than other systemic symptoms.	5: 42%<br>4: 58%<br>3: 0%<br>2: 0%<br>1: 0%	Consensus reached
**Key question 2: Which diagnostic tests are recommended for achieving an accurate diagnosis?**		
The histopathological diagnosis of cHL must be established according to the WHO classification criteria, preferably on an excisional lymph node biopsy. There are four subtypes of cHL: nodular sclerosis, mixed cellularity, lymphocyte-rich, and lymphocyte-depleted. These subtypes differ in terms of clinical presentation, sites of involvement, epidemiology, and association with Epstein-Barr virus, although their management is largely similar. Nodular lymphocyte-predominant HL remains a distinct pathological, biological, and clinical entity.	5: 84%<br>4: 16%<br>3: 0%<br>2: 0%<br>1: 0%	Consensus reached
The diagnostic work-up for HL should include a thorough medical history, physical examination, and assessment of B symptoms. Imaging studies must comprise chest X-ray, total-body CT with and without contrast, and total-body PET. Laboratory tests should include ESR, comprehensive metabolic panel, and viral serologies. A pregnancy test is mandatory for women of childbearing age, along with fertility counseling for young patients. Cardiac and pulmonary assessments, including electrocardiography, echocardiography, and spirometry, are also recommended prior to initiating therapy.	5: 89%<br>4: 11%<br>3: 0%<br>2: 0%<br>1: 0%	Consensus reached
Fine-needle aspiration biopsy may be inadequate for the diagnosis of HL, as only the examination of an intact lymph node architecture provides the detailed information necessary for an accurate histopathological diagnosis.	5: 84%<br>4: 11%<br>3: 5%<br>2: 0%<br>1: 0%	Consensus reached
Fine-needle biopsy may serve as an alternative when excisional biopsy is not technically feasible.	5: 63%<br>4: 32%<br>3: 5%<br>2: 0%<br>1: 0%	Consensus reached
**Key Question 3: Which medical specialists should be involved in the diagnostic process?**		
A multidisciplinary approach is essential to ensure accurate diagnosis and personalized treatment for patients with HL.	5: 68%<br>4: 26%<br>3: 6%	Consensus reached

	2: 0%<br>1: 0%	
The specialists who should be involved in the diagnostic phase include the surgeon for performing lymph node and/or extranodal biopsies, the pathologist for histological diagnosis, the radiologist for staging and mapping pathological lymph nodes, and the nuclear medicine physician for performing and interpreting PET-CT scans.	5: 84%<br>4: 11%<br>3: 5%<br>2: 0%<br>1: 0%	Consensus reached
**Key Question 4: Which prognostic factors should be taken into account?**		
For early-stage (I–IA) HL, prognostic factors have been well established and include a mediastinum-to-thorax ratio >0.35, ESR >50 in the absence of B symptoms or ESR >30 in the presence of B symptoms, involvement of multiple lymph node regions, presence of extranodal sites, age >50 years, and extensive splenic involvement. These criteria are supported by major cooperative groups such as European Organisation for Research and Treatment of Cancer, German Hodgkin Study Group, and National Comprehensive Cancer Network.	5: 78%<br>4: 17%<br>3: 5%<br>2: 0%<br>1: 0%	Consensus reached
For advanced-stage HL, several prognostic scoring systems have been developed. One of the most widely used is the International Prognostic Factors Project score, which incorporates seven adverse variables: age >45 years, stage IV disease, male sex, white blood cell count >15,000/mm^3, lymphocyte count <600/mm^3, serum albumin <4 g/dL, and hemoglobin <10.5 g/dL. Each factor scores 1 point, with the total score correlating with progressively worse prognosis.	5: 89%<br>4: 11%<br>3: 0%<br>2: 0%<br>1: 0%	Consensus reached
Staging is the primary prognostic indicator in cHL and is essential to guide therapeutic planning. Key factors to be evaluated include the presence of supradiaphragmatic and/or infradiaphragmatic lymphadenopathy, the number of involved nodal sites, the presence of bulky nodal disease, contiguous or disseminated extranodal involvement, and the presence of B symptoms.	5: 83%<br>4: 17%<br>3: 0%<br>2: 0%<br>1: 0%	Consensus reached
Patients with cHL presenting with five or more risk factors have a 5-year PFS rate of approximately 42%, whereas those with no risk factors exhibit a 5-year PFS rate of around 84%.	5: 61%<br>4: 28%<br>3: 6%<br>2: 0%<br>1: 5%	Consensus reached
**Key Question 5: What is the optimal therapeutic approach according to the stage of the disease?**		
First-line treatment for newly diagnosed cHL is guided by clinical stage and prognostic risk factors, following a risk-adapted approach. Interim FDG PET imaging after two cycles of chemotherapy is pivotal in modulating treatment intensity. A negative PET result may allow for de-escalation strategies, such as reducing the number of cycles, omitting bleomycin, or administering lower doses of RT. Conversely, a positive interim PET may indicate the need for treatment intensification. A positive EOT PET scan can guide the use of consolidative RT on residual disease sites. The introduction of BV in first-line therapy, especially for advanced-stage disease, has been associated with a reduced prognostic impact of interim FDG PET in terms of both PFS and overall survival.	5: 59%<br>4: 35%<br>3: 6%<br>2: 0%<br>1: 0%	Consensus reached
Initial treatment for patients with HL is tailored to individual risk factors, taking into account histologic subtype, anatomical stage, presence of B symptoms, and the occurrence of bulky disease (defined as a mass >10 cm).	5: 61%<br>4: 39%<br>3: 0%<br>2: 0%<br>1: 0%	Consensus reached

First-line therapy for favorable early-stage disease (I–IIA): two cycles of ABVD plus involved-node RT (20 Gy).<br>First-line therapy for unfavorable early-stage disease: four cycles of ABVD plus involved-field RT (IFRT, 30 Gy) or two cycles of escalated BEACOPP plus two cycles of ABVD plus involved-field RT (30 Gy).<br>First-line therapy for advanced-stage disease (IIB–IV): two cycles of ABVD; if FDG-PET is negative, proceed with four cycles of AVD.<br>If FDG-PET is positive, intensify treatment with four cycles of escalated BEACOPP.<br>In advanced-stage cHL (stage IV), two cycles of BV-AVD are followed by four cycles (if FDG-PET is negative).<br>Primary refractory disease (5–10%): proceed immediately to ASCT after debulking therapy.<br>Relapsed disease (20–30%): in eligible patients, high-dose chemotherapy, stem cell collection and ASCT.<br>Classical high-dose chemotherapy regimens (ICE, BEGEV, DHAP, etc.) or regimens using anti-CD30 BV plus chemotherapy.<br>Patients with risk factors for relapse after ASCT may receive consolidation therapy with anti-CD30 BV.<br>ASCT + reduced-intensity conditioning allo-HSCT or haploidentical HSCT in patients with multiple relapses.<br>Anti-PD-1 agents (pembrolizumab or nivolumab vs BV) have shown significant efficacy and safety in patients relapsed after ASCT or allo-HSCT.	5: 67%<br>4: 28%<br>3: 5%<br>2: 0%<br>1: 0%	Consensus reached
Older patients or those with comorbidities (cardiac, renal), as well as pregnant patients, require personalized therapeutic approaches.	5: 72%<br>4: 28%<br>3: 0%<br>2: 0%<br>1: 0%	Consensus reached
For patients with advanced-stage HL, nivolumab (anti-PD-1) combined with AVD may become the new standard of care, offering better tolerability than BV-AVD and reducing the need for RT.	5: 58%<br>4: 29%<br>3: 13%<br>2: 0%<br>1: 0%	Consensus reached
**Key Question 6: Which criteria should be applied to assess treatment response?**		
Interim PET is essential for determining whether to maintain, adjust, or complete the treatment plan, enabling tailored therapy based on the patient's response.	5: 78%<br>4: 22%<br>3: 0%<br>2: 0%<br>1: 0%	Consensus reached
A positive EOT PET scan, evaluated using the DS, may warrant consolidative therapy (such as RT) or a change in the therapeutic strategy.	5: 83%<br>4: 17%<br>3: 0%<br>2: 0%<br>1: 0%	Consensus reached
A positive EOT PET scan predicts a higher risk of relapse than CT imaging alone.	5: 76%<br>4: 12%<br>3: 6%<br>2: 0%<br>1: 6%	Consensus reached
A positive interim PET scan may indicate the need for treatment intensification.	5: 35%<br>4: 41%<br>3: 18%<br>2: 6%<br>1: 0%	Consensus reached

A positive PET scan at any stage of treatment requires a repeat biopsy to confirm active disease.	5: 50%<br>4: 33%<br>3: 6%<br>2: 11%<br>1: 0%	Consensus reached
**Key Question 7: Which clinical visits and diagnostic tests are required for appropriate follow-up, and how frequently should they be conducted?**		
Long-term attention should be paid to the potential cardiopulmonary toxicities resulting from treatment.	5: 89%<br>4: 11%<br>3: 0%<br>2: 0%<br>1: 0%	Consensus reached
It is advisable to perform periodic CT scans every 6 months for at least 2 years to monitor for potential relapses.	5: 67%<br>4: 17%<br>3: 11%<br>2: 5%<br>1: 0%	Consensus reached
Clinical evaluation, medical history, and blood tests (including activity markers, such as ESR) should be performed every 3 months during the first 6 months, every 6 months for the following 4 years, and then annually.	5: 59%<br>4: 29%<br>3: 6%<br>2: 6%<br>1: 0%	Consensus reached
Female patients with HL who received subaxillary irradiation before the age of 40 years should undergo annual mammography starting 8–10 years after the completion of RT. Those irradiated before the age of 30 should also undergo breast MRI as part of the surveillance protocol.	5: 56%<br>4: 33%<br>3: 11%<br>2: 0%<br>1: 0%	Consensus reached
Thyroid-stimulating hormone levels should be assessed annually in patients with HL who have received neck RT.	5: 71%<br>4: 29%<br>3: 0%<br>2: 0%<br>1: 0%	Consensus reached
In young patients with HL undergoing intensive therapies, testosterone or estrogen levels should be monitored.	5: 41%<br>4: 35%<br>3: 11%<br>2: 0%<br>1: 0%	Consensus reached
**Key Question 8: What salvage treatment options are recommended for relapsed or refractory disease?**		
ASCT remains the main salvage treatment for patients with relapsed or refractory HL.	5: 83%<br>4: 11%<br>3: 6%<br>2: 0%<br>1: 5%	Consensus reached
The introduction of BV and anti-PD-1 agents has improved outcomes in patients with relapsed/refractory HL following ASCT.	5: 94%<br>4: 6%<br>3: 0%<br>2: 0%<br>1: 0%	Consensus reached
The use of BV and anti-PD-1 agents in first-line treatment introduces challenges in the sequencing of therapeutic options for patients with high-risk or advanced-stage HL, highlighting the need for strategic treatment planning from the time of diagnosis.	5: 67%<br>4: 28%<br>3: 5%<br>2: 0%<br>1: 0%	Consensus reached

Consensus was defined based on a predefined agreement threshold (≥75%), calculated by summing the percentages of responses scoring 4

(agreement) and 5 (full agreement), as indicated by participants on a 5-point Likert scale (1 = complete disagreement; 5 = complete agreement).
ABVD: doxorubicin, bleomycin, vinblastine and dacarbazine; ASCT: autologous stem cell transplant; allo-HSCT: allogeneic hematopoietic stem cell transplantation; AVD: doxorubicin, vinblastine, and dacarbazine; BEACOPP: bleomycin sulfate, etoposide phosphate, doxorubicin hydrochloride, cyclophosphamide, vincristine sulfate, procarbazine hydrochloride, and prednisone; BEGEV: bendamustin, gemcitabine, vinorelbine; BV: brentuximab vedotin; BV-AVD: brentuximab vedotin, doxorubicin, vinblastine, and dacarbazine; cHL: classical Hodgkin lymphoma; DHAP: dexamethasone, high-dose cytarabine and cisplatin; EOT: end-of-treatment; ESR: erythrocyte sedimentation rate; FDG: fludeoxyglucose; HL: Hodgkin lymphoma; HSCT: hematopoietic stem cell transplantation; ICE: ifosfamide, carboplatin and etoposide; PFS: progression-free survival; RT: radiotherapy.

Supplementary Table 2. Results from the Delphi process on the clinical management of diffuse large B-cell lymphoma.

**Key question 1: Which clinical signs and symptoms may suggest the presence of the disease?**		
Statements	***Consensus (%)***	***Decision***
Signs that may raise suspicion of the disease include a usually painless enlargement of one or more superficial lymph node stations, laterocervical swelling, organomegaly (hepatosplenomegaly), asthenia, fever, profuse night sweats, unexplained weight loss exceeding 10% of total body weight over the past 6 months, and pruritus of unknown origin.	5: 80%<br>4: 15%<br>3: 5%<br>2: 0%<br>1: 0%	Consensus reached
Imaging studies, such as ultrasound, can reveal organomegaly or focal lesions.	5: 60%<br>4: 30%<br>3: 10%<br>2: 0%<br>1: 0%	Consensus reached
Additional symptoms are associated with lymphoma involvement in specific anatomical sites, such as the CNS, skin, gastrointestinal tract, and gonads.	5: 55%<br>4: 40%<br>3: 5%<br>2: 0%<br>1: 0%	Consensus reached
Hematochemical abnormalities may also be detected, including lymphocytosis, elevated calcium levels, and signs of bone marrow failure, such as anemia and thrombocytopenia.	5: 50%<br>4: 30%<br>3: 20%<br>2: 0%<br>1: 0%	Consensus reached
**Key question 2: Which diagnostic tests are recommended for achieving an accurate diagnosis?**		
An excisional or incisional biopsy of the affected lymph node or tissue is required for diagnostic purposes. In cases of nodal involvement, excisional biopsies are preferred over core needle biopsies, as the latter are generally suboptimal because of their limited ability to provide sufficient tissue for both histopathological evaluation and ancillary studies. In patients with significant comorbidities, multiple core needle biopsies using a 16- or 18-gauge needle at different sites within the lymph node may be considered.	5: 95%<br>4: 5%<br>3: 0%<br>2: 0%<br>1: 0%	Consensus reached
Regardless of the approach used, obtaining adequate and sufficient tissue for proper immunohistochemical and molecular characterization is essential. Histopathological evaluation should include immunophenotypic analysis through IHC, assessing markers as recommended by the 2022 WHO classification, including CD45, CD20, CD19, and/or CD79a,	5: 90%<br>4: 10%<br>3: 0%<br>2: 0%<br>1: 0%	Consensus reached

PAX5, CD3, CD5, BCL6, CD10, BCL2, c-MYC, Ki-67, and IRF4/MUM1.		
The presence of high-grade cytology, strong MYC expression (>40%) and BCL2 expression (>50%), and the GCB subtype warrants fluorescence in situ hybridization analysis for *MYC* and *BCL2* gene rearrangements. Detection of these rearrangements leads to the diagnosis of DLBCL or high-grade B-cell lymphoma with MYC and BCL2 rearrangements, according to the 2022 WHO classification.	5: 90%<br>4: 5%<br>3: 5%<br>2: 0%<br>1: 0%	Consensus reached
The 2022 WHO and 2022 ICC classifications recommend maintaining the distinction between GCB and non-GCB/activated B-cell subtypes in cases of DLBCL not otherwise specified. This differentiation should be performed using an immunohistochemical algorithm, specifically the Hans classifier, which relies on three markers: CD10, BCL6, and MUM1.	5: 65%<br>4: 30%<br>3: 5%<br>2: 0%<br>1: 0%	Consensus reached
In cases of suspected gastric involvement, endoscopic biopsy with systematic gastric mucosal mapping and *Helicobacter pylori* testing is recommended.	5: 60%<br>4: 35%<br>3: 5%<br>2: 0%<br>1: 0%	Consensus reached
For lymphomas with CNS involvement, cerebrospinal fluid analysis is essential, along with contrast-enhanced brain MRI.	5: 80%<br>4: 20%<br>3: 0%<br>2: 0%<br>1: 0%	Consensus reached
In cases of deep retroperitoneal/abdominal or thoracic lymphadenopathy that is not easily accessible surgically, patients should be considered for exploratory laparoscopy/laparotomy or mediastinoscopy/video-assisted thoracoscopic surgery. If a surgical approach is not feasible, a core biopsy using a Tru-Cut needle under CT or ultrasound guidance may be performed.	5: 75%<br>4: 20%<br>3: 5%<br>2: 0%<br>1: 0%	Consensus reached
Required laboratory tests include complete blood count, erythrocyte sedimentation rate, serum lactate dehydrogenase, renal and liver function tests, and protein profile. Further evaluation should include immunoglobulin levels, albumin, uric acid, beta-2 microglobulin, prothrombin time, activated partial thromboplastin time, and fibrinogen. Circulating lymphocyte immunophenotyping is recommended in cases where a leukemic phase of the disease is suspected. Serological testing for Epstein-Barr virus, HBV (anti-HBc total antibodies, with HBV-DNA quantification if positive), HCV, and HIV should also be performed.	5: 85%<br>4: 15%<br>3: 0%<br>2: 0%<br>1: 0%	Consensus reached
Contrast-enhanced whole-body CT and whole-body 18F-FDG PET are essential for diagnosis and staging.	5: 80%<br>4: 15%<br>3: 0%<br>2: 5%<br>1: 0%	Consensus reached
A thorough physical examination and detailed medical history should be conducted, along with an assessment of Performance Status (PS). In older patients, geriatric rating scales should be used to assess overall fitness and treatment tolerance.	5: 79%<br>4: 16%<br>3: 5%<br>2: 0%<br>1: 0%	Consensus reached
In cases of pulmonary involvement where CT-guided biopsy is not advisable, bronchoscopy with biopsy may be considered as an alternative approach.	5: 40%<br>4: 40%<br>3: 20%<br>2: 0%	Consensus reached

	1: 0%	
In selected cases, assessment of specific deficiencies, such as glucose-6-phosphate dehydrogenase deficiency, may be clinically relevant.	5: 42%<br>4: 16%<br>3: 32%<br>2: 5%<br>1: 5%	Consensus not reached
In certain cases, bone biopsy may serve as an alternative to lymph node biopsy.	5: 21%<br>4: 16%<br>3: 42%<br>2: 16%<br>1: 5%	Consensus not reached
**Key Question 3: Which medical specialists should be involved in the diagnostic process?**		
Specialists involved in the diagnostic process include surgeons (general surgeons, otolaryngologists, neurosurgeons, and interventional radiologists) and pathologists for histological diagnosis.	5: 80%<br>4: 15%<br>3: 5%<br>2: 0%<br>1: 0%	Consensus reached
Additionally, depending on the specific clinical needs, it would be beneficial to involve molecular biologists, pulmonologists, gastroenterologists, palliative care specialists, nuclear medicine physicians, dermatologists, cardiologists, flow cytometrists, geneticists, interventional radiologists, psychotherapists, gynecologists/andrologists, urologists, diabetologists, and psychologists.	5: 42%<br>4: 53%<br>3: 5%<br>2: 0%<br>1: 0%	Consensus reached
For older patients, consultation with a geriatrician or a cardio-oncologist is recommended.	5: 39%<br>4: 39%<br>3: 22%<br>2: 0%<br>1: 0%	Consensus reached
**Key Question 4: Which prognostic factors should be taken into account?**		
Prognostic indicators to consider include histology with IHC (GC vs. non-GC), the IPI, the age-adjusted IPI (for patients under 60 years), the Revised IPI, and the CNS-IPI, which helps identify patients at high risk of CNS progression/relapse who may benefit from CNS prophylactic therapy.	5: 84%<br>4: 16%<br>3: 0%<br>2: 0%<br>1: 0%	Consensus reached
Molecular factors, such as *c-MYC*, *BCL2*, and *BCL6* gene mutations, have significant prognostic value and can influence treatment decisions, particularly in cases of double-hit lymphoma and triple-hit lymphoma.	5: 85%<br>4: 15%<br>3: 0%<br>2: 0%<br>1: 0%	Consensus reached
Other factors include bulky disease status and patient age, both of which impact the IPI. Additionally, diagnostic and therapeutic lumbar puncture may be required in cases of CNS involvement.	5: 65%<br>4: 35%<br>3: 0%<br>2: 0%<br>1: 0%	Consensus reached
The activated B-cell-DLBCL subtype, according to cell of origin classification, is associated with an unfavorable prognosis.	5: 63%<br>4: 37%<br>3: 0%<br>2: 0%<br>1: 0%	Consensus reached
The DLBCL/high-grade B-cell lymphoma with MYC and BCL2 rearrangements represents a prognostically unfavorable molecular subgroup, demonstrating reduced responsiveness to conventional therapies.	5: 80%<br>4: 20%<br>3: 0%<br>2: 0%<br>1: 0%	Consensus reached
**Key Question 5: What is the optimal therapeutic approach according to the stage of the disease?**		

For patients with IPI 3–5, the polatuzumab-rituximab-CHP regimen is preferred.	5: 74%<br>4: 21%<br>3: 5%<br>2: 0%<br>1: 0%	Consensus reached
For Burkitt lymphoma, more intensive chemotherapy protocols are used, including R-BFM, Magrath, or R-Hyper-CVAD.	5: 90%<br>4: 10%<br>3: 0%<br>2: 0%<br>1: 0%	Consensus reached
In primary CNS lymphomas, treatment includes high-dose MTX and ARA-C, with ASCT as a potential consolidation strategy.	5: 90%<br>4: 10%<br>3: 0%<br>2: 0%<br>1: 0%	Consensus reached
In advanced-stage (stage III–IV) disease with IPI >1, treatment consists of six cycles of R-CHOP plus two additional infusions of rituximab.	5: 74%<br>4: 11%<br>3: 10%<br>2: 5%<br>1: 0%	Consensus reached
In high-risk cases, such as double-hit or triple-hit lymphomas, Dose-adjusted EPOCH-R is commonly used, although no universally accepted standard therapy exists.	5: 74%<br>4: 21%<br>3: 5%<br>2: 0%<br>1: 0%	Consensus reached
For early-stage (I/II) disease with IPI 0, four cycles of R-CHOP plus two additional infusions of rituximab are recommended.	5: 63%<br>4: 32%<br>3: 5%<br>2: 0%<br>1: 0%	Consensus reached
Patients with aggressive non-HLs and HIV who have viral load controlled by antiretroviral therapy are treated using the same regimens as HIV-negative patients.	5: 84%<br>4: 16%<br>3: 0%<br>2: 0%<br>1: 0%	Consensus reached
For older or frail patients, R-CVP or R-mini-CHOP are the preferred treatment options, while in patients with cardiac comorbidities, the use of liposomal anthracyclines is recommended.	5: 68%<br>4: 21%<br>3: 0%<br>2: 11%<br>1: 0%	Consensus reached
Post-transplant lymphoproliferative disorders should initially be managed with IS reduction, in combination with rituximab with or without CHT.	5: 68%<br>4: 32%<br>3: 0%<br>2: 0%<br>1: 0%	Consensus reached
The therapeutic strategy varies based on disease stage.	5: 58%<br>4: 21%<br>3: 21%<br>2: 0%<br>1: 0%	Consensus reached
**Key Question 6: Which criteria should be applied to assess treatment response?**		
The final treatment response is assessed using PET/CT, performed at least 4–6 weeks after treatment completion, according to the Lugano response criteria with DS evaluation.	5: 78%<br>4: 17%<br>3: 5%<br>2: 0%<br>1: 0%	Consensus reached

An interim evaluation at the third or fourth cycle using CT and, when indicated, PET/CT, may help identify primary non-responders, allowing early initiation of second-line therapy (CAR-T).	5: 84%<br>4: 16%<br>3: 0%<br>2: 0%<br>1: 0%	Consensus reached
The final evaluation includes a physical examination, laboratory tests, CT, and PET/CT scans.	5: 84%<br>4: 16%<br>3: 0%<br>2: 0%<br>1: 0%	Consensus reached
In cases of positive initial findings, a bone, gastric, or colon biopsy should be performed as needed.	5: 74%<br>4: 16%<br>3: 5%<br>2: 5%<br>1: 0%	Consensus reached
**Key Question 7: Which clinical visits and diagnostic tests are required for appropriate follow-up, and how frequently should they be conducted?**		
PET/CT is reserved for cases of suspected relapse and is not routinely used in follow-up, except in specific scenarios, such as bone involvement.	5: 79%<br>4: 11%<br>3: 5%<br>2: 5%<br>1: 0%	Consensus reached
A physical examination is recommended, along with ultrasound in selected cases, mammography screening, a Pap test for women, and an evaluation for signs of post-treatment toxicity.	5: 61%<br>4: 33%<br>3: 6%<br>2: 0%<br>1: 0%	Consensus reached
In patients with cardiac disease, cardiologic assessments, including electrocardiography and echocardiography, are recommended every 2 years for those who have received anthracyclines.	5: 74%<br>4: 21%<br>3: 0%<br>2: 0%<br>1: 5%	Consensus reached
During the first 2 years, laboratory tests should be performed every 3 months, alternating CT scans with ultrasound of superficial and abdominal lymph nodes. After this period, the frequency of evaluations decreases, with one CT scan per year and an ultrasound twice a year for the following 2 years. Beyond the second year, clinical visits and evaluations can be conducted every 6 months, maintaining annual CT scans.	5: 35%<br>4: 35%<br>3: 6%<br>2: 12%<br>1: 12%	Consensus not reached
**Key Question 8: What salvage treatment options are recommended for relapsed or refractory disease?**		
As third-line therapy, CAR-T therapy (axi-cel or tisa-cel) is considered for eligible patients who have not received bendamustine-containing regimens in the previous 6 months.	5: 67%<br>4: 22%<br>3: 6%<br>2: 0%<br>1: 5%	Consensus reached
Bispecific monoclonal antibodies, such as glofitamab or epcoritamab, can be used as third-line therapy. In patients who are ineligible for CAR-T therapy or transplantation, alternative options include R-Pola-Benda), or Tafa-Lena, particularly for older or unfit patients.	5: 85%<br>4: 10%<br>3: 5%<br>2: 0%<br>1: 0%	Consensus reached
As second-line therapy, in cases of early relapse within 12 months, CAR-T therapy (axi-cel) should be considered for eligible patients, with rapid management toward lymphocyte apheresis. Salvage therapy consists of R-DHAP followed by ASCT for patients who are ineligible for CAR-T therapy or, if CAR-T is unavailable, but are eligible for transplantation. For	5: 85%<br>4: 15%<br>3: 0%<br>2: 0%<br>1: 0%	Consensus reached

patients who are ineligible for both CAR-T and transplantation, alternative options include Tafa-Lena or R-Pola-Benda.		
As second-line therapy for late relapse (after 12 months), salvage therapy with R-DHAP (2–4 cycles) followed by ASCT is recommended for transplant-eligible patients. For those who are ineligible for transplantation, alternative treatment options include Tafa-Lena or R-Pola-Benda.	5: 79%<br>4: 21%<br>3: 0%<br>2: 0%<br>1: 0%	Consensus reached
As third-line therapy, allo-HSCT may be a suitable option for eligible patients experiencing relapse after CAR-T therapy.	5: 74%<br>4: 21%<br>3: 0%<br>2: 0%<br>1: 5%	Consensus reached
As third-line therapy, palliative approaches should be considered for patients who are ineligible for aggressive treatments.	5: 63%<br>4: 32%<br>3: 0%<br>2: 5%<br>1: 0%	Consensus reached

Consensus was defined based on a predefined agreement threshold (≥75%), calculated by summing the percentages of responses scoring 4 (agreement) and 5 (full agreement), as indicated by participants on a 5-point Likert scale (1 = complete disagreement; 5 = complete agreement).
ARA-C: Cytarabine; allo-HSCT: allogeneic hematopoietic stem cell transplantation; ASCT: autologous stem cell transplant; CHT: chemotherapy; CAR-T: chimeric antigen receptor T-cell; CNS: central nervous system; DLBCL: diffuse large B-cell lymphoma; DS: Deauville Score; EPOCH-R: etoposide, prednisone, vincristine, cyclophosphamide, doxorubicin, and rituximab; FDG: fludeoxyglucose; GC: Germinal Center; GCB: germinal center B-cell; HBV: hepatitis B virus; HCV: hepatitis C virus; IS: immunosuppression; HL: Hodgkin lymphoma; IHC: immunohistochemistry; IPI: International Prognostic Index; MTX: methotrexate; R-BFM: revised-Berlin-Frankfurt-Münster; R-CHOP: rituximab, cyclophosphamide, doxorubicin, vincristine, and prednisone; R-CHP: Rituximab - Cyclophosphamide - Doxorubicin – Prednisone; R-CVP: Rituximab - Cyclophosphamide – Vincristine **-** Prednisone; R-Hyper-CVAD: Rituximab - Hyper-fractionated Cyclophosphamide, Vincristine, Adriamycin (Doxorubicina), and Dexamethasone; R-mini-CHOP: variante attenuata del classico R-CHOP; R-DHAP: rituximab, dexamethasone, cytarabine, cisplatin; R-Pola-Benda: rituximab, polatuzumab vedotin, bendamustine; Tafa-Lena: tafasitamab-lenalidomide.

Supplementary Table 3. Results from the Delphi process on the clinical management of follicular lymphoma.

**Key question 1: Which clinical signs and symptoms may suggest the presence of the disease?**		
Statements	***Consensus (%)***	***Decision***
FL may present with either localized or generalized lymphadenopathy. In some cases, splenomegaly may also be observed.	5: 84%<br>4: 16%<br>3: 0%<br>2: 0%<br>1: 0%	Consensus reached
In advanced stages, the disease may lead to systemic symptoms (B symptoms), such as weight loss, fever, and night sweats, as well as pruritus.	5: 84%<br>4: 11%<br>3: 5%<br>2: 0%<br>1: 0%	Consensus reached
In cases with marked bone marrow infiltration, FL may present with cytopenias and related symptoms, such as fatigue and infections.	5: 79%<br>4: 21%<br>3: 0%	Consensus reached

	2: 0%<br>1: 0%	
As with other B-cell lymphoproliferative neoplasms, autoimmune manifestations may be observed in rare cases, most commonly autoimmune hemolytic anemia and immune thrombocytopenia.	5: 68%<br>4: 21%<br>3: 11%<br>2: 0%<br>1: 0%	Consensus reached
In rare cases, FL may originate from extranodal sites and present with symptoms related to the site of infiltration (e.g., dyspepsia, indigestion, or occult bleeding in primary duodenal FL).	5: 67%<br>4: 28%<br>3: 5%<br>2: 0%<br>1: 0%	Consensus reached
**Key question 2: Which diagnostic tests are recommended for achieving an accurate diagnosis?**		
The diagnosis of FL is established through a biopsy of the affected lymph node or involved tissue. In cases of lymph node involvement, excisional or incisional biopsies are preferred over core needle biopsy.	5: 84%<br>4: 16%<br>3: 0%<br>2: 0%<br>1: 0%	Consensus reached
If patients have comorbidities that contraindicate lymph node biopsy, multiple core needle biopsies using a 16- or 18-gauge needle may be performed as an alternative.	5: 79%<br>4: 16%<br>3: 0%<br>2: 5%<br>1: 0%	Consensus reached
Histological examination should include immunophenotypic analysis by IHC, in accordance with the 2022 WHO classification.	5: 84%<br>4: 16%<br>3: 0%<br>2: 0%<br>1: 0%	Consensus reached
The immunophenotypic profile of FL is typically: CD20+, CD10+, BCL2+, CD23+/–, CD5–, BCL6+ and/or LMO2+. Occasionally, FL may be CD10– or BCL2–.	5: 84%<br>4: 16%<br>3: 0%<br>2: 0%<br>1: 0%	Consensus reached
In 85–90% of FL, a BCL2/IGH rearrangement – t(14;18)(q32;q21) – is detected by FISH, leading to immunohistochemical overexpression of the BCL2 protein. This finding supports the diagnosis of FL. Testing for this rearrangement is recommended in the diagnostic work-up of FLs that are immunonegative for BCL2 expression by IHC and to aid in the differential diagnosis from other low-grade B-cell lymphomas, such as marginal zone lymphoma. In this context, evaluation of the *BCL6* gene translocation is also recommended, further supporting the diagnosis of FL.	5: 84%<br>4: 16%<br>3: 0%<br>2: 0%<br>1: 0%	Consensus reached
In high-grade forms of FL that are negative for CD10 expression and lack *BCL2* gene rearrangement, the diagnostic work-up should include immunohistochemical assessment of the IRF4/MUM1 marker. High expression of this marker correlates with *IRF4* (MUM1) gene rearrangement. This finding would support a diagnosis of IRF4 (MUM1)-rearranged large B-cell lymphoma.	5: 79%<br>4: 21%<br>3: 0%<br>2: 0%<br>1: 0%	Consensus reached
In high-grade FL, grade 3B (International Consensus Classification)/follicular large B-cell lymphoma, as defined by the 2022 WHO classification and closely related to diffuse large B-cell lymphoma, it is important to assess MYC protein expression. If MYC expression is elevated (>40%), testing for *MYC* gene rearrangement is recommended to exclude the possibility of	5: 84%<br>4: 16%<br>3: 0%<br>2: 0%<br>1: 0%	Consensus reached

progression to a high-grade lymphoma with dual BCL2 and MYC rearrangements.		
Duodenal-type FL is a distinct entity typically localized to the small intestine. Its morphology, immunophenotype, and genetic features are similar to those of nodal FL grade 1–2. However, most patients present with clinically indolent and localized disease.	5: 72%<br>4: 28%<br>3: 0%<br>2: 0%<br>1: 0%	Consensus reached
Inguinal-onset forms more frequently exhibit a diffuse growth pattern and a 1p36 gene deletion detected by FISH analysis. They typically lack BCL2 rearrangement and express the CD23 marker on IHC.	5: 74%<br>4: 26%<br>3: 0%<br>2: 0%<br>1: 0%	Consensus reached
Flow cytometric analysis of peripheral blood can identify leukemic-phase cases.	5: 74%<br>4: 26%<br>3: 0%<br>2: 0%<br>1: 0%	Consensus reached
Staging of FL, according to the Lugano criteria, involves the following assessments: PET/CT scans, contrast-enhanced CT of the neck, chest, abdomen, and pelvis, bone marrow biopsy, and evaluation of B symptoms – defined as weight loss >10% of baseline body weight, night sweats, and fever >38°C. Additional investigations may be required based on specific symptoms or suspected extranodal involvement, such as gastroscopy and endoscopic ultrasound in cases of duodenal lymphoma.	5: 83%<br>4: 11%<br>3: 56<br>2: 0%<br>1: 0%	Consensus reached
Additional evaluations include comprehensive blood tests such as complete blood count, serum LDH, beta-2 microglobulin, serologic markers for HBV, HCV, and HIV, liver and kidney function tests, electrolytes, total protein, serum protein electrophoresis, immunoglobulin quantification, and uric acid levels. A 2D echocardiogram, electrocardiography, and a cardiology consultation are also recommended prior to initiating chemotherapy or immunotherapy.	5: 83%<br>4: 17%<br>3: 0%<br>2: 0%<br>1: 0%	Consensus reached
Additional useful assessments include evaluation for gamete cryopreservation in women of childbearing age and men up to 50 years old prior to the initiation of chemotherapy; placement of a central venous access device (e.g., peripherally inserted central catheter line or port-à-cath) when indicated for patients undergoing chemotherapy; and a thorough vaccination history.	5: 89%<br>4: 11%<br>3: 0%<br>2: 0%<br>1: 0%	Consensus reached
**Key Question 3: Which medical specialists should be involved in the diagnostic process?**		
The management of FL requires a multidisciplinary approach involving hematologists, pathologists, radiologists, radiation oncologists, nuclear medicine physicians, and molecular biologists.	5: 84%<br>4: 16%<br>3: 0%<br>2: 0%<br>1: 0%	Consensus reached
The pathologist plays a central role in the diagnosis and classification of the disease, ensuring that the biopsy specimen is adequate for accurate evaluation. Collaboration with nuclear medicine physicians and radiologists is essential for the interpretation of diagnostic imaging, which is crucial for accurate staging and assessment of treatment response according to the Lugano criteria.	5: 78%<br>4: 22%<br>3: 0%<br>2: 0%<br>1: 0%	Consensus reached
In certain cases, it may be necessary to involve additional specialists, such as an infectious disease specialist for the prophylaxis of occult HBV infection and the management of infectious complications, or a cardiologist for pre-treatment cardio-oncologic assessment.	5: 74%<br>4: 21%<br>3: 5%<br>2: 0%<br>1: 0%	Consensus reached

It is advisable that individual cases – especially the more complex ones – be discussed within a dedicated multidisciplinary team focused on lymphoproliferative neoplasms.	5: 83%<br>4: 17%<br>3: 0%<br>2: 0%<br>1: 0%	Consensus reached
**Key Question 4: Which prognostic factors should be taken into account?**		
The FLIPI and FLIPI-2 are validated tools for assessing prognosis in patients with newly diagnosed FL. FLIPI is based on age, Ann Arbor stage, number of involved nodal sites, hemoglobin level, and LDH level. FLIPI-2 includes age, hemoglobin level, the largest diameter of the involved lymph node, beta-2 microglobulin level, and bone marrow involvement.	5: 83%<br>4: 17%<br>3: 0%<br>2: 0%<br>1: 0%	Consensus reached
Recent studies have shown that baseline total metabolic tumor volume assessed by PET/CT and response to induction therapy assessed by end-of-treatment PET/CT with a DS of 1–3 are prognostic predictors in FL.	5: 89%<br>4: 11%<br>3: 0%<br>2: 0%<br>1: 0%	Consensus reached
Progression of disease within 24 months from initial treatment, which affects approximately 15% of patients requiring therapy at diagnosis, is a well-established negative prognostic indicator.	5: 89%<br>4: 11%<br>3: 0%<br>2: 0%<br>1: 0%	Consensus reached
Minimal residual disease, assessed by real-time PCR on peripheral blood or bone marrow (in diagnosis-positive cases), has a documented prognostic value but is not yet routinely used in clinical practice. A potential surrogate is multiparametric flow cytometry performed on peripheral blood and bone marrow in patients with detectable disease at baseline.	5: 83%<br>4: 11%<br>3: 6%<br>2: 0%<br>1: 0%	Consensus reached
Prognostic models incorporating molecular biology to predict outcomes, such as the m7-FLIPI, are not used routinely in clinical practice.	5: 83%<br>4: 11%<br>3: 6%<br>2: 0%<br>1: 0%	Consensus reached
**Key Question 5: What is the optimal therapeutic approach according to the stage of the disease?**		
In patients with localized disease (stage I or stage II with contiguous lymph nodes), involved-site radiotherapy (24 Gy) targeting the initial disease sites is the recommended therapeutic strategy.	5: 89%<br>4: 11%<br>3: 0%<br>2: 0%<br>1: 0%	Consensus reached
In cases of localized disease (stage I or stage II with contiguous lymph nodes) where involved-site radiotherapy is contraindicated, monotherapy with the anti-CD20 monoclonal antibody rituximab may be employed as an alternative treatment option.	5: 89%<br>4: 11%<br>3: 0%<br>2: 0%<br>1: 0%	Consensus reached
For patients with stage II disease with non-contiguous lymph nodes or advanced-stage disease (stage III–IV) with low tumor burden, clinical observation (watch-and-wait) represents the optimal management strategy.	5: 78%<br>4: 22%<br>3: 0%<br>2: 0%<br>1: 0%	Consensus reached
In patients with advanced-stage disease, high tumor burden, and meeting GELF criteria for treatment initiation, chemoimmunotherapy is the recommended therapeutic approach.	5: 89%<br>4: 11%<br>3: 0%<br>2: 0%<br>1: 0%	Consensus reached
Induction treatments with rituximab-bendamustine for six cycles and R-CHOP for six cycles are considered first-line therapeutic options.	5: 88%<br>4: 12%<br>3: 0%	Consensus reached

	2: 0%<br>1: 0%	
Treatment with R-CHOP may be preferred in cases where histological transformation is suspected but cannot be confirmed by biopsy. In patients with a history of cardiac disease, substituting conventional doxorubicin with liposomal doxorubicin should be considered.	5: 89%<br>4: 11%<br>3: 0%<br>2: 0%<br>1: 0%	Consensus reached
Rituximab may be replaced with obinutuzumab in combination with bendamustine or CHOP in patients with intermediate- to high-risk FLIPI scores.	5: 89%<br>4: 11%<br>3: 0%<br>2: 0%<br>1: 0%	Consensus reached
In patients who achieve a complete or partial metabolic response on end-of-treatment PET/CT, maintenance therapy with the anti-CD20 monoclonal antibody used during induction (rituximab or obinutuzumab) is recommended, administered every 2 months for a total of 12 doses.	5: 89%<br>4: 11%<br>3: 0%<br>2: 0%<br>1: 0%	Consensus reached
In patients over 80 years of age or those considered frail due to comorbidities, a personalized treatment approach is appropriate, using lower-toxicity regimens such as rituximab monotherapy, reduced-dose bendamustine, or R-CVP.	5: 83%<br>4: 17%<br>3: 0%<br>2: 0%<br>1: 0%	Consensus reached
**Key Question 6: Which criteria should be applied to assess treatment response?**		
Treatment response should be assessed using PET/CT with application of the DS.	5: 83%<br>4: 17%<br>3: 0%<br>2: 0%<br>1: 0%	Consensus reached
The Lugano criteria are used to classify treatment response and include the evaluation of PET/CT and CT imaging performed 4 to 6 weeks after completion of therapy.	5: 89%<br>4: 11%<br>3: 0%<br>2: 0%<br>1: 0%	Consensus reached
In patients with bone marrow involvement at diagnosis, a post-treatment bone marrow biopsy is recommended to assess response.	5: 83%<br>4: 17%<br>3: 0%<br>2: 0%<br>1: 0%	Consensus reached
**Key Question 7: Which clinical visits and diagnostic tests are required for appropriate follow-up, and how frequently should they be conducted?**		
Follow-up for patients in complete response may be conducted every 3 months during the first year, then every 6 months until the fifth year, and annually thereafter.	5: 83%<br>4: 17%<br>3: 0%<br>2: 0%<br>1: 0%	Consensus reached
Clinical follow-up is recommended and should include a complete physical examination and laboratory tests.	5: 83%<br>4: 17%<br>3: 0%<br>2: 0%<br>1: 0%	Consensus reached
Surveillance imaging with CT may be performed at most every 6 months during the first 2 years following completion of treatment, and subsequently no more than once per year (or as clinically indicated).	5: 78%<br>4: 17%<br>3: 0%<br>2: 5%<br>1: 0%	Consensus reached
PET/CT is not recommended for routine follow-up in patients who are in complete response.	5: 82%<br>4: 18%	Consensus reached

	3: 0%<br>2: 0%<br>1: 0%	
In the years following treatment, it is important to include monitoring for potential late toxicities related to oncologic therapies (e.g., cardiotoxicity, myelodysplastic syndromes) as well as screening for second primary malignancies.	5: 83%<br>4: 17%<br>3: 0%<br>2: 0%<br>1: 0%	Consensus reached
Ultrasound examinations of the entire abdomen and the lymph nodes in the neck, axillary, and inguinal regions may complement the clinical evaluation.	5: 72%<br>4: 17%<br>3: 11%<br>2: 0%<br>1: 0%	Consensus reached
The patient may also be monitored within dedicated lymphoma survivorship clinics over the subsequent 5–10 years.	5: 89%<br>4: 11%<br>3: 0%<br>2: 0%<br>1: 0%	Consensus reached
**Key Question 8: What salvage treatment options are recommended for relapsed or refractory disease?**		
Patients with relapsed disease often benefit from a period of clinical observation. Disease relapse should be histologically confirmed, particularly in the presence of elevated LDH, non-homogeneous lymph node growth, extranodal involvement, bulky disease >7 cm, or the onset of systemic symptoms. Areas with high SUVmax on PET/CT (especially SUVmax >13) are suspicious for histological transformation and should be targeted for biopsy. The decision to initiate therapy should be based on the same GELF criteria used at initial diagnosis.	5: 83%<br>4: 17%<br>3: 0%<br>2: 0%<br>1: 0%	Consensus reached
At each relapse requiring treatment, consideration should be given to enrolling the patient in a clinical trial.	5: 78%<br>4: 17%<br>3: 5%<br>2: 0%<br>1: 0%	Consensus reached
In patients with first relapse within 24 months of initial treatment, particularly in the presence of bulky disease and high SUVmax on PET/CT, and who are eligible for transplantation, the potential benefit of salvage chemotherapy followed by autologous hematopoietic stem cell transplantation should be considered and compared with a chemo-free using the R2 regimen (rituximab-lenalidomide).	5: 94%<br>4: 6%<br>3: 0%<br>2: 0%<br>1: 0%	Consensus reached
In patients with late relapse (beyond 24 months) or those not eligible for transplantation, a chemo-free approach with the R2 regimen is preferred.	5: 89%<br>4: 11%<br>3: 0%<br>2: 0%<br>1: 0%	Consensus reached
In cases of second or third relapse, treatment options may include bispecific antibody therapy with mosunetuzumab, the R2 regimen if not previously used, or CAR-T therapy (axi-cel, tisa-cel). The choice between bispecific antibodies and CAR-T therapy remains an area of ongoing clinical debate.	5: 89%<br>4: 11%<br>3: 0%<br>2: 0%<br>1: 0%	Consensus reached
With regard to supportive therapies, primary prophylaxis with G-CSF should be considered in older patients or those with bone marrow infiltration; antimicrobial prophylaxis with acyclovir and trimethoprim-sulfamethoxazole should be evaluated during induction chemotherapy; antiviral prophylaxis is recommended for patients with occult HBV infection; and HCV eradication should be undertaken either at the time of FL diagnosis or after	5: 94%<br>4: 6%<br>3: 0%<br>2: 0%<br>1: 0%	Consensus reached

completion of induction therapy, depending on disease burden and urgency of oncologic treatment.		

Consensus was defined based on a predefined agreement threshold (≥75%), calculated by summing the percentages of responses scoring 4 (agreement) and 5 (full agreement), as indicated by participants on a 5-point Likert scale (1 = complete disagreement; 5 = complete agreement).
FISH: fluorescence in situ hybridization; FL: Follicular lymphoma; FLIPI: Follicular Lymphoma International Prognostic Index; G-CSC: granulocyte colony-stimulating factor; GELF: Groupe d'Étude des Lymphomes Folliculaires; HBV: hepatitis B virus; IHC: immunohistochemistry; LDH: lactate dehydrogenase; R-CHOP: rituximab, cyclophosphamide, doxorubicin, vincristine, and prednisone; R-CVP: Rituximab - Cyclophosphamide – Vincristine - Prednisone.

Supplementary Table 4. Results from the Delphi process on the clinical management of mantle cell lymphoma.

**Key question 1: Which clinical signs and symptoms may suggest the presence of the disease?**		
Statements	***Consensus (%)***	***Decision***
The clinical signs that may raise suspicion of MCL include hematologic abnormalities, such as lymphocytosis, thrombocytopenia, or anemia, often associated with bone marrow involvement.	5: 63%<br>4: 32%<br>3: 5%<br>2: 0%<br>1: 0%	Consensus reached
The signs suggestive of MCL largely depend on the anatomical sites involved by the disease.	5: 63%<br>4: 32%<br>3: 5%<br>2: 0%<br>1: 0%	Consensus reached
The signs that may raise suspicion of MCL include superficial or deep lymphadenopathies, which may exert compressive effects on internal organs or vascular structures.	5: 63%<br>4: 21%<br>3: 11%<br>2: 5%<br>1: 0%	Consensus reached
The signs suggestive of MCL include those related to extranodal involvement, which may be present even at disease onset, such as involvement of the gastrointestinal tract, nasopharynx, lung or kidney.	5: 63%<br>4: 37%<br>3: 0%<br>2: 0%<br>1: 0%	Consensus reached
Secondary symptoms that may raise suspicion of MCL include fever, fatigue and pruritus.	5: 42%<br>4: 37%<br>3: 16%<br>2: 5%<br>1: 0%	Consensus reached
Among the signs that may raise suspicion of MCL is the presence of hemolytic anemia.	5: 37%<br>4: 37%<br>3: 26%<br>2: 0%<br>1: 0%	Consensus NOT reached
**Key question 2: Which diagnostic tests are recommended for achieving an accurate diagnosis?**		
The diagnosis of MCL is established through lymph node biopsy or biopsy of the involved tissue. In cases of lymph node involvement, excisional or incisional biopsies are preferred over fine-needle aspiration, as the latter may be suboptimal for diagnostic adequacy, ancillary studies (including	5: 74%<br>4: 21%<br>3: 5%<br>2: 0%<br>1: 0%	Consensus reached

immunohistochemistry and molecular analyses), and the assessment of prognostic biomarkers.		
In patients with comorbidities, multiple core needle biopsies using a 16- or 18-gauge needle may be performed at different sites within the lymph node.	5: 63%<br>4: 32%<br>3: 0%<br>2: 0%<br>1: 5%	Consensus reached
Histological examination should include immunophenotypic analysis, as determined by IHC investigation.	5: 79%<br>4: 21%<br>3: 0%<br>2: 0%<br>1: 0%	Consensus reached
The immunophenotypic profile of MCL is characterized by CD20+, CD79a+, CD19+, CD5+, cyclin D1+, IgM+, IgD+, and SOX11+, and CD10-, CD23-, BCL6-, and CD43-. Light chain restriction is typically lambda-positive with kappa-negative or weak expression.	5: 89%<br>4: 11%<br>3: 0%<br>2: 0%<br>1: 0%	Consensus reached
The hallmark genetic abnormality of MCL is the t(11;14)(q13;q32) translocation, present in over 95% of cases. This translocation leads to overexpression and hyperactivation of cyclin D1, a key regulator of the cell cycle, which can be demonstrated by IHC.	5: 89%<br>4: 11%<br>3: 0%<br>2: 0%<br>1: 0%	Consensus reached
The majority of MCL cases express the transcription factor SOX11. Rare SOX11-negative cases are associated with a more indolent clinical course, more frequent leukemic presentation, reduced nodal involvement, and a lower likelihood of disease progression.	5: 79%<br>4: 21%<br>3: 0%<br>2: 0%<br>1: 0%	Consensus reached
The 2022 WHO classification of hematolymphoid neoplasms and the 2022 International Consensus Classification classification identify three subtypes of MCL: classical MCL (nodal or extranodal), non-nodal leukemic MCL, and in situ mantle cell neoplasia.	5: 89%<br>4: 11%<br>3: 0%<br>2: 0%<br>1: 0%	Consensus reached
From a cytological perspective, four variants of MCL are recognized: blastoid, pleomorphic, small cell, and marginal zone-like.	5: 95%<br>4: 5%<br>3: 0%<br>2: 0%<br>1: 0%	Consensus reached
The blastoid variant of MCL should be considered in the differential diagnosis of acute leukemias and other aggressive subtypes of non-HLs.	5: 89%<br>4: 11%<br>3: 0%<br>2: 0%<br>1: 0%	Consensus reached
High-grade cytological variants of MCL may lack SOX11 expression; therefore, analysis of the t(11;14)(q13;q32) translocation is essential for differential diagnosis, particularly to distinguish MCL from diffuse large B-cell lymphoma that aberrantly express cyclin D1.	5: 79%<br>4: 21%<br>3: 0%<br>2: 0%<br>1: 0%	Consensus reached

The pathologist plays a central role in the diagnosis of the disease by integrating morphological assessment with immunophenotypic profiling and molecular data.	5: 89%<br>4: 11%<br>3: 0%<br>2: 0%<br>1: 0%	Consensus reached
**Key Question 3: Which medical specialists should be involved in the diagnostic process?**		
In the initial phase, otolaryngologists, general surgeons, or, less commonly, thoracic surgeons may be involved, depending on the anatomical location of the disease.	5: 61%<br>4: 28%<br>3: 11%<br>2: 0%<br>1: 0%	Consensus reached
The involvement of medical specialists varies according to the location of the lymphadenopathy.	5: 69%<br>4: 26%<br>3: 5%<br>2: 0%<br>1: 0%	Consensus reached
Cardiology consultation should be included for specific evaluations, particularly in patients scheduled to undergo complex treatment regimens.	5: 68%<br>4: 32%<br>3: 0%<br>2: 0%<br>1: 0%	Consensus reached
In selected cases (localized disease), the involvement of radiation oncologists is advisable.	5: 53%<br>4: 42%<br>3: 0%<br>2: 5%<br>1: 0%	Consensus reached
In the presence of extranodal manifestations, it is essential to involve the appropriate specialist based on the site of involvement.	5: 48%<br>4: 42%<br>3: 0%<br>2: 10%<br>1: 0%	Consensus reached
**Key Question 4: Which prognostic factors should be taken into account?**		
Compared with the classical variant, blastoid morphology is associated with an unfavorable prognosis.	5: 89%<br>4: 11%<br>3: 0%<br>2: 0%<br>1: 0%	Consensus reached
A Ki-67 proliferation index greater than 30% is recognized to be associated with a more aggressive clinical course.	5: 79%<br>4: 21%<br>3: 0%<br>2: 0%<br>1: 0%	Consensus reached
Genetic abnormalities involving the *TP53* gene (mutations and/or deletions) are associated with poor prognosis.	5: 89%<br>4: 11%<br>3: 0%<br>2: 0%<br>1: 0%	Consensus reached
In younger patients, *TP53* gene mutations are associated with inferior responses to conventional treatment.	5: 89%<br>4: 11%<br>3: 0%<br>2: 0%<br>1: 0%	Consensus reached
TP53 gene mutation, assessed by sequencing analysis, is a key prognostic marker that, when identified in classical MCL, supports the selection of appropriate treatment strategies.	5: 79%<br>4: 21%<br>3: 0%<br>2: 0%	Consensus reached

	1: 0%	
TP53 gene mutations are generally associated with strong nuclear expression of the p53 immunohistochemical marker in more than 50% of tumor cells.	5: 68%<br>4: 32%<br>3: 0%<br>2: 0%<br>1: 0%	Consensus reached
The prognostic model used for MCL is the Mantle Cell Lymphoma International Prognostic Index.	5: 79%<br>4: 21%<br>3: 0%<br>2: 0%<br>1: 0%	Consensus reached
SOX11 positivity is associated with more aggressive forms of the disease.	5: 42%<br>4: 58%<br>3: 0%<br>2: 0%<br>1: 0%	Consensus reached
The immunoglobulin mutational status may serve as a prognostic indicator.	5: 21%<br>4: 37%<br>3: 26%<br>2: 16%<br>1: 0%	Consensus NOT reached
**Key Question 5: What is the optimal therapeutic approach according to the stage of the disease?**		
In aggressive forms of the disease, treatment is stratified based on age and patient fitness. In younger, fit patients, a combination regimen (e.g., R-CHOP alternating with R-DHAP) is used, followed by autologous stem cell transplant and maintenance therapy with rituximab for 3 years. In older patients (over 65 years) or those considered unfit, a less intensive approach, such as rituximab plus bendamustine followed by rituximab maintenance for 3 years, is preferred.	5: 72%<br>4: 22%<br>3: 6%<br>2: 0%<br>1: 0%	Consensus reached
In fit older patients, the treatment of choice is the R-BAC regimen.	5: 76%<br>4: 18%<br>3: 6%<br>2: 0%<br>1: 0%	Consensus reached
The indolent form does not require immediate treatment but involves regular monitoring every 3 months.	5: 67%<br>4: 28%<br>3: 0%<br>2: 5%<br>1: 0%	Consensus reached
Maintenance therapy with rituximab may be omitted in selected patients, such as those at high risk of infection.	5: 56%<br>4: 39%<br>3: 5%<br>2: 0%<br>1: 0%	Consensus reached
Particular attention should be paid to accurately characterizing fit older patients who may be eligible for more intensive therapy.	5: 83%<br>4: 17%<br>3: 0%<br>2: 0%<br>1: 0%	Consensus reached
**Key Question 6: Which criteria should be applied to assess treatment response?**		
Response assessment is primarily based on repeating the investigations performed at disease onset, including CT and PET scans to evaluate disease burden and metabolic activity, as well as endoscopic examinations to assess the persistence or resolution of disease in the involved organs.	5: 94%<br>4: 6%<br>3: 0%<br>2: 0%<br>1: 0%	Consensus reached

In leukemic forms, bone marrow evaluation is essential for assessing treatment response.	5: 90%<br>4: 5%<br>3: 5%<br>2: 0%<br>1: 0%	Consensus reached
In addition to imaging studies, clinical evaluation is also important, particularly in patients who initially presented with prominent symptoms.	5: 83%<br>4: 17%<br>3: 0%<br>2: 0%<br>1: 0%	Consensus reached
**Key Question 7: Which clinical visits and diagnostic tests are required for appropriate follow-up, and how frequently should they be conducted?**		
Follow-up includes clinical evaluations every 3 to 6 months during the first year, along with CT scans every 6 months during the first 2 years.	5: 83%<br>4: 11%<br>3: 0%<br>2: 6%<br>1: 0%	Consensus reached
PET imaging is essential at the end of treatment and subsequently in cases where residual disease is suspected based on CT findings.	5: 89%<br>4: 11%<br>3: 0%<br>2: 0%<br>1: 0%	Consensus reached
**Key Question 8: What salvage treatment options are recommended for relapsed or refractory disease?**		
The current standard of care for relapsed MCL is treatment with a BTKi, both in younger and older patients.	5: 89%<br>4: 11%<br>3: 0%<br>2: 0%<br>1: 0%	Consensus reached
In both younger and fit older patients, following progression on a BTKi, the current treatment of choice is CAR-T therapy or allogeneic stem cell transplantation. Allogeneic transplantation is considered in patients who are ineligible for CAR-T therapy, in cases of logistical barriers to CAR-T administration, or in the event of relapse after CAR-T therapy.	5: 83%<br>4: 17%<br>3: 0%<br>2: 0%<br>1: 0%	Consensus reached
In older or unfit patients, non-covalent BTKi therapy may be considered following disease progression.	5: 89%<br>4: 11%<br>3: 0%<br>2: 0%<br>1: 0%	Consensus reached
It is advisable to perform a biopsy before initiating second-line treatment to assess *TP53* mutational status and Ki-67 proliferation index.	5: 55%<br>4: 33%<br>3: 6%<br>2: 0%<br>1: 6%	Consensus reached
Early referral to specialized centers capable of independently administering CAR-T therapy is essential.	5: 78%<br>4: 11%<br>3: 11%<br>2: 0%<br>1: 0%	Consensus reached
The importance of early planning of potential patient eligibility for CAR-T therapy should be emphasized.	5: 89%<br>4: 11%<br>3: 0%<br>2: 0%<br>1: 0%	Consensus reached
Close monitoring is essential in patients with adverse prognostic factors, with early reevaluation if appropriate.	5: 67%<br>4: 28%<br>3: 5%	Consensus reached

	2: 0%<br>1: 0%	

Consensus was defined based on a predefined agreement threshold (≥75%), calculated by summing the percentages of responses scoring 4 (agreement) and 5 (full agreement), as indicated by participants on a 5-point Likert scale (1 = complete disagreement; 5 = complete agreement).
BTKi: Bruton tyrosine kinase inhibitor; CAR-T: chimeric antigen receptor T-cell; HL: Hodgkin lymphoma; IHC: immunohistochemistry; MCL: mantle cell lymphoma; R-CHOP: rituximab, cyclophosphamide, doxorubicin, vincristine, and prednisone; R-DHAP: rituximab, dexamethasone, cytarabine, cisplatin; R-BAC: rituximab, bendamustine and cytarabine.

Supplementary Table 5. Results from the Delphi process on the clinical management of peripheral T-cell lymphomas and breast implant-associated anaplastic large cell lymphoma.

**Key question 1: Which clinical signs and symptoms may suggest the presence of the disease?**		
Statements	***Consensus (%)***	***Decision***
The main systemic symptoms of T-cell lymphoma include lymphadenopathy, which may be localized or generalized. In some cases, other organs may also be involved, such as the liver, spleen, lungs, or intestines.	5: 79%<br>4: 21%<br>3: 0%<br>2: 0%<br>1: 0%	Consensus reached
B symptoms include weight loss (≥10% of body weight over the past 6 months), unexplained fever (>37.5 °C for at least 1 week), and night sweats.	5: 84%<br>4: 16%<br>3: 0%<br>2: 0%<br>1: 0%	Consensus reached
In some cases, PTCLs may involve extranodal sites and present with symptoms related to the infiltration site, such as abdominal or chest pain or dyspnea.	5: 89%<br>4: 11%<br>3: 0%<br>2: 0%<br>1: 0%	Consensus reached
In cases with bone marrow involvement, PTCLs may also present with cytopenias and related symptoms, such as fatigue and infections.	5: 89%<br>4: 11%<br>3: 0%<br>2: 0%<br>1: 0%	Consensus reached
**Key question 2: Which diagnostic tests are recommended for achieving an accurate diagnosis?**		
The diagnosis of T-cell lymphoma is established through biopsy of involved lymph nodes or other affected tissues. In cases of nodal involvement, excisional biopsies are preferred over needle biopsies, as the latter are generally considered suboptimal for diagnostic accuracy because of their limited ability to provide sufficient tissue for comprehensive analysis, including ancillary studies. In patients with significant comorbidities, multiple core needle biopsies using a 16- or 18-gauge needle at different sites within the lymph node may be performed as an alternative approach.	5: 84%<br>4: 16%<br>3: 0%<br>2: 0%<br>1: 0%	Consensus reached
Histopathological evaluation must include immunophenotypic analysis through immunohistochemistry staining, with marker assessment according to the 2022 WHO classification. The recommended panel includes: CD20, CD3, CD10, BCL6, Ki-67, CD5, CD30, CD2, CD4, CD8, CD7, CD56, CD21, CD23, TCRβ, TCRδ, PD1/CD279, ALK, and TP63.	5: 84%<br>4: 16%<br>3: 0%<br>2: 0%<br>1: 0%	Consensus reached
To characterize specific subtypes of PTCL, it is necessary to evaluate markers of the cell of origin, including TFH cell markers	5: 89%<br>4: 11%<br>3: 0%	Consensus reached

(CD10, BCL6, PD1/CD279, ICOS, and CXCL13), as well as cytotoxic T-cell markers (TIA-1, granzyme B, and/or perforin).	2: 0%<br>1: 0%	
Another essential marker is Epstein-Barr encoding region in situ hybridization for the detection of Epstein-Barr virus.	5: 79%<br>4: 21%<br>3: 0%<br>2: 0%<br>1: 0%	Consensus reached
Under certain circumstances, molecular clonality testing is required to assess *TCRG* gene rearrangements.	5: 84%<br>4: 16%<br>3: 0%<br>2: 0%<br>1: 0%	Consensus reached
In cases of ALK-negative ALCL, it is important to consider cytogenetic analysis (FISH) to detect *DUSP22* gene rearrangements. Additionally, if there is immunohistochemical expression of the TP63 marker, testing for *TP63* gene rearrangement should also be considered.	5: 63%<br>4: 37%<br>3: 0%<br>2: 0%<br>1: 0%	Consensus reached
Another ancillary investigation is flow cytometry, which employs a panel of markers including CD45, CD3, CD5, CD19, CD10, CD20, CD30, CD4, CD8, CD7, CD2, TCRα, and TCRβ, along with TCRγ analysis, for the diagnosis and staging of PTCL. This analysis can be performed on samples obtained from peripheral blood, pleural effusions, ascitic fluid, and cerebrospinal fluid.	5: 79%<br>4: 21%<br>3: 0%<br>2: 0%<br>1: 0%	Consensus reached
BMB is recommended for disease staging. Histological analysis of the BMB allows for the detection of bone marrow involvement by atypical lymphoid populations, supported by immunohistochemical studies and supplemented, when appropriate, by flow cytometric and molecular clonality analyses. The latter are typically performed on aspirated material.	5: 89%<br>4: 11%<br>3: 0%<br>2: 0%<br>1: 0%	Consensus reached
This diagnostic approach is used to define disease entities with preferential bone marrow involvement, such as T-cell prolymphocytic leukemia, T-cell large granular lymphocytic leukemia, natural killer cell large granular lymphocyte leukemia, aggressive natural killer cell leukemia, adult T-cell leukemia/lymphoma, and hepatosplenic T-cell lymphoma.	5: 79%<br>4: 16%<br>3: 5%<br>2: 0%<br>1: 0%	Consensus reached
**Key Question 3: Which medical specialists should be involved in the diagnostic process?**		
The diagnosis of T-cell lymphoma requires a multidisciplinary approach involving hematologists, pathologists, surgeons, flow cytometry specialists, cardio-oncologists, radiologists, and nuclear medicine physicians.	5: 84%<br>4: 16%<br>3: 0%<br>2: 0%<br>1: 0%	Consensus reached
A multidisciplinary team approach is preferable, involving hematopathologists, hematologists, and/or oncologists, radiation oncologists, and other specialists – ideally with expertise in PTCLs.	5: 78%<br>4: 17%<br>3: 5%<br>2: 0%<br>1: 0%	Consensus reached
The pathologist plays a crucial role in the diagnosis and classification of T-cell lymphomas.	5: 89%<br>4: 11%<br>3: 0%<br>2: 0%<br>1: 0%	Consensus reached

In certain cases, the involvement of additional specialists, such as infectious disease experts, cardiologists, endocrinologists, or neurologists, may be required.	5: 68%<br>4: 26%<br>3: 6%<br>2: 0%<br>1: 0%	Consensus reached
**Key Question 4: Which prognostic factors should be taken into account?**		
The International Prognostic Index (IPI), although originally developed for aggressive B-cell lymphomas, is also adapted for prognostic assessment in T-cell lymphomas and is strongly influenced by disease stage and extent.	5: 83%<br>4: 17%<br>3: 0%<br>2: 0%<br>1: 0%	Consensus reached
The IPI, originally developed for aggressive lymphomas, has proven to be an effective prognostic tool for nodal PTCL. Although other more specific prognostic scores for PTCL-NOS have been proposed, none has demonstrated superiority over the IPI. Therefore, in clinical practice, the IPI remains the preferred prognostic tool.	5: 83%<br>4: 17%<br>3: 0%<br>2: 0%<br>1: 0%	Consensus reached
The PINK score (including age >60 years, stage III or IV disease, distant lymph node involvement, and extranasal disease) and the PINK-E score (which adds the presence of detectable Epstein-Barr virus DNA at diagnosis) are key prognostic indicators, particularly for NK/T-cell lymphomas and ENKTCL.	5: 78%<br>4: 17%<br>3: 5%<br>2: 0%<br>1: 0%	Consensus reached
Other relevant prognostic indicators include geriatric assessment, the modified PIT, and the IPI.	5: 78%<br>4: 22%<br>3: 0%<br>2: 0%<br>1: 0%	Consensus reached
Disease extent and staging are the main prognostic indicators, along with elevated levels of lactate dehydrogenase and beta-2 microglobulin, potential skin or bone involvement, and the presence of biological markers that distinguish different subtypes, such as ALK-positive and ALK-negative ALCL.	5: 78%<br>4: 22%<br>3: 0%<br>2: 0%<br>1: 0%	Consensus reached
**Key Question 5: What is the optimal therapeutic approach according to the stage of the disease?**		
PTCL-NOS, AITL and TFH lymphoma		
CHOEP, CHOP, or CHOP-like regimens may be offered as first-line induction therapy.	5: 82%<br>4: 12%<br>3: 6%<br>2: 0%<br>1: 0%	Consensus reached
Consolidative Rt (e.g., 30–40 Gy) may be considered for patients with early-stage disease (stage I–II) who achieve a response following CHOP or CHOP-like chemotherapy.	5: 81%<br>4: 19%<br>3: 0%<br>2: 0%<br>1: 0%	Consensus reached
Consolidative ASCT should be considered in responding patients with either limited or advanced-stage disease.	5: 83%<br>4: 17%<br>3: 0%<br>2: 0%<br>1: 0%	Consensus reached
ALCL		
Consolidative ASCT should be considered in responding patients with either limited or advanced-stage disease.	5: 83%<br>4: 17%<br>3: 0%<br>2: 0%<br>1: 0%	Consensus reached
In patients with limited-stage, non-bulky ALK-positive ALCL and a favorable pre-treatment risk profile, chemotherapy alone (BV-	5: 83%<br>4: 17%	Consensus reached

CHP) may be sufficient. Patients who do not respond should subsequently be managed according to recommendations for high-risk ALK-positive ALCL.	3: 0%<br>2: 0%<br>1: 0%	
For patients with ALK-negative ALCL who are chemo-sensitive and eligible for transplant, BV-CHP followed by consolidative ASCT in first complete resonse/partial response is recommended.	5: 83%<br>4: 17%<br>3: 0%<br>2: 0%<br>1: 0%	Consensus reached
Consolidative RT (e.g., 30–40 Gy in 15–20 fractions) may be considered for limited-stage ALCL.	5: 71%<br>4: 29%<br>3: 0%<br>2: 0%<br>1: 0%	Consensus reached
ASCT cannot be routinely recommended for patients with ALK-positive ALCL. However, it may be considered on a case-by-case basis in patients with particularly high-risk features, such as an IPI score of 3–4, bulky extranodal disease, or suspected but unconfirmed residual disease activity.	5: 78%<br>4: 22%<br>3: 0%<br>2: 0%<br>1: 0%	Consensus reached
HSCTL		
In the absence of clinical trials, fit patients who are eligible for transplantation should be offered a more intensive chemotherapy regimen than CHOP.	5: 78%<br>4: 22%<br>3: 0%<br>2: 0%<br>1: 0%	Consensus reached
ICE is suggested as the preferred induction regimen. Other options include IVAC, DHAP, DHAOx, CHOEP, and dose-adjusted EPOCH.	5: 89%<br>4: 11%<br>3: 0%<br>2: 0%<br>1: 0%	Consensus reached
Chemotherapy options for frail or unfit patients, and/or those ineligible for transplantation, include GEMOX, reduced-dose ICE, and dose-adjusted EPOCH.	5: 67%<br>4: 33%<br>3: 0%<br>2: 0%<br>1: 0%	Consensus reached
Response assessment with PET/CT should be supported by bone marrow biopsy. In some cases, liver biopsy may also be required.	5: 66%<br>4: 28%<br>3: 6%<br>2: 0%<br>1: 0%	Consensus reached
When feasible, bone marrow and peripheral blood samples should be analyzed by flow cytometry to assess tumor cell surface antigens that are not reliably detectable by routine immunohistochemistry (e.g., CD52).	5: 61%<br>4: 28%<br>3: 11%<br>2: 0%<br>1: 0%	Consensus reached
Eligible patients who respond to treatment (complete response or partial response) should undergo consolidative HSCT, preferably allo-HSCT. Autologous HSCT is recommended for patients who are not eligible for allogeneic transplantation.	5: 71%<br>4: 18%<br>3: 12%<br>2: 0%<br>1: 0%	Consensus reached
Responding patients (complete or partial response) should preferably undergo allo-SCT as consolidative therapy. Autologous transplantation is recommended for patients who are not eligible for an allogeneic transplantation.	5: 76%<br>4: 18%<br>3: 6%<br>2: 0%<br>1: 0%	Consensus reached
ENKTCL		
Epstein-Barr virus DNA in peripheral blood should be monitored using quantitative PCR at baseline and during treatment as a	5: 56%<br>4: 39%	Consensus reached

biomarker of response, in addition to imaging-based response assessment.	3: 6%<br>2: 0%<br>1: 0%	
Fit patients with limited-stage disease should receive RT (≥50 Gy) combined with concomitant, intercalated, or sequential chemotherapy that is anthracycline-free and includes L-asparaginase (e.g., DDGP or mSMILE for transplant-eligible patients, and AspMetDex or P-GEMOX for transplant-ineligible patients).	5: 78%<br>4: 22%<br>3: 0%<br>2: 0%<br>1: 0%	Consensus reached
An L-asparaginase–containing regimen may be offered to patients with stage III–IV nasal disease or stage I–IV extranasal disease (e.g., DDGP or mSMILE for transplant-eligible patients, and AspMetDex or P-GEMOX for transplant-ineligible patients). The addition of involved-site RT may be considered on a case-by-case basis.	5: 71%<br>4: 29%<br>3: 0%<br>2: 0%<br>1: 0%	Consensus reached
In high-risk responding patients who are eligible for transplantation, consolidative autologous or allo-SCT may be considered. The choice of transplantation should be made on a case-by-case basis, considering several factors: pre-treatment risk profile, response to first-line therapy, Eastern Cooperative Oncology Group performance status, and donor availability.	5: 76%<br>4: 24%<br>3: 0%<br>2: 0%<br>1: 0%	Consensus reached
EATL		
Fit, transplant-eligible patients may be offered first-line therapy consisting of one cycle of CHOP or CHOEP, followed by three cycles of IVE alternating with intermediate-dose MTX. Alternative regimens include CHOEP or dose-adjusted EPOCH.	5: 72%<br>4: 22%<br>3: 6%<br>2: 0%<br>1: 0%	Consensus reached
Consolidation with ASCT may be recommended for responding patients.	5: 72%<br>4: 28%<br>3: 0%<br>2: 0%<br>1: 0%	Consensus reached
Frail or unfit patients, and/or those ineligible for transplantation, may be offered six cycles of CHOP or CHOP-like chemotherapy.	5: 72%<br>4: 28%<br>3: 0%<br>2: 0%<br>1: 0%	Consensus reached
T-LGL and NK-LGL		
Asymptomatic patients without severe cytopenias or significant splenomegaly may initially be managed with observation alone.	5: 72%<br>4: 28%<br>3: 0%<br>2: 0%<br>1: 0%	Consensus reached
Symptomatic patients (with anemia and/or transfusion-dependent thrombocytopenia, severe neutropenia [neutrophils $<0.5\times10^9$/L], and/or symptomatic splenomegaly) may initiate first-line immunosuppressive therapy with low-dose weekly methotrexate (preferred in patients with associated autoimmune disease), cyclophosphamide (with or without steroids), or cyclosporine A (the latter two preferred in patients with severe cytopenias).	5: 72%<br>4: 28%<br>3: 0%<br>2: 0%<br>1: 0%	Consensus reached
The efficacy of first-line treatment can be assessed after 3–4 months, and the same regimen may be continued if the response (complete or partial) and treatment tolerability are satisfactory.	5: 78%<br>4: 22%<br>3: 0%<br>2: 0%<br>1: 0%	Consensus reached
MEITL		

Treatment options for transplant-eligible patients include ICE (preferred), IVAC, DHAP, DHAX, CHOEP, and dose-adjusted EPOCH.	5: 72%<br>4: 28%<br>3: 0%<br>2: 0%<br>1: 0%	Consensus reached
Treatment options for transplant-ineligible patients include GEMOX, reduced-dose ICE, and GDP.	5: 72%<br>4: 28%<br>3: 0%<br>2: 0%<br>1: 0%	Consensus reached
Consolidative allo-HSCT may be considered in chemo-sensitive patients (complete or partial response) who are eligible for transplant and have a suitable donor.	5: 67%<br>4: 33%<br>3: 0%<br>2: 0%<br>1: 0%	Consensus reached
ATLL		
Asymptomatic indolent ATLL can be managed with active surveillance without immediate therapeutic intervention.	5: 78%<br>4: 22%<br>3: 0%<br>2: 0%<br>1: 0%	Consensus reached
Patients with acute or lymphoma-type ATLL with bulky disease may be treated with hyper-CVAD, CHOP, CHOEP, or dose-adjusted EPOCH.	5: 78%<br>4: 22%<br>3: 0%<br>2: 0%<br>1: 0%	Consensus reached
Patients who achieve a response may proceed to allo-SCT.	5: 72%<br>4: 28%<br>3: 0%<br>2: 0%<br>1: 0%	Consensus reached
Human T-lymphotropic virus 1 testing may be offered to first-degree relatives and partners of patients diagnosed with ATLL.	5: 72%<br>4: 28%<br>3: 0%<br>2: 0%<br>1: 0%	Consensus reached
All patients may be offered antimicrobial prophylaxis against opportunistic infections. In the case of positive serology for *Strongyloides stercoralis*, treatment may be initiated even in asymptomatic individuals.	5: 78%<br>4: 22%<br>3: 0%<br>2: 0%<br>1: 0%	Consensus reached
CNS prophylaxis may be recommended for all patients with acute or lymphoma-type ATLL.	5: 83%<br>4: 17%<br>3: 0%<br>2: 0%<br>1: 0%	Consensus reached
BIA-ALCL		
Total capsulectomy with removal of the breast implant and excision of any associated mass is recommended for patients with no evidence of further disease spread.	5: 67%<br>4: 33%<br>3: 0%<br>2: 0%<br>1: 0%	Consensus reached
If suspicious regional lymph nodes are identified, a representative biopsy – preferably excisional – should be performed.	5: 72%<br>4: 28%<br>3: 0%<br>2: 0%<br>1: 0%	Consensus reached

Removal of the contralateral implant is recommended, particularly if it is textured.	5: 72%<br>4: 28%<br>3: 0%<br>2: 0%<br>1: 0%	Consensus reached
Mastectomy cannot be recommended.	5: 61%<br>4: 33%<br>3: 6%<br>2: 0%<br>1: 0%	Consensus reached
Six cycles of BV-CHP, CHOP, CHOEP, or dose-adjusted EPOCH are recommended for patients with residual disease after involved-site RT and those with advanced-stage disease (stage III–IV).	5: 67%<br>4: 33%<br>3: 0%<br>2: 0%<br>1: 0%	Consensus reached
RT (e.g., 30 Gy) is recommended following surgery in patients with TNM-adapted stage IIA–IIB disease and in those with limited-stage disease if residual disease is evident.	5: 67%<br>4: 33%<br>3: 0%<br>2: 0%<br>1: 0%	Consensus reached
ASCT may be considered in patients who respond to chemotherapy.	5: 65%<br>4: 24%<br>3: 6%<br>2: 5%<br>1: 0%	Consensus reached
Bilateral capsulectomy may be considered in responding patients with advanced disease following chemotherapy.	5: 71%<br>4: 29%<br>3: 6%<br>2: 5%<br>1: 0%	Consensus reached
For all PTCLs, patients should be enrolled in a clinical trial whenever possible.	5: 61%<br>4: 28%<br>3: 11%<br>2: 5%<br>1: 0%	Consensus reached
**Key Question 6: Which criteria should be applied to assess treatment response?**		
Treatment response is assessed through imaging evaluation according to the Lugano criteria and is based on disease staging and regression. Patients are classified as being in complete remission, partial remission, having no response, or experiencing disease progression.	5: 83%<br>4: 17%<br>3: 0%<br>2: 0%<br>1: 0%	Consensus reached
PET and/or CT should be performed prior to treatment and during restaging, particularly at the end of induction.	5: 89%<br>4: 11%<br>3: 0%<br>2: 0%<br>1: 0%	Consensus reached
Some studies have reported lower sensitivity of PET/CT in detecting bone marrow involvement in PTCL compared with Hodgkin lymphoma and diffuse large B-cell lymphoma.	5: 67%<br>4: 33%<br>3: 0%<br>2: 0%<br>1: 0%	Consensus reached
Bone marrow examination may reveal associated myeloid disorders, such as underlying clonal hematopoiesis, which is frequently observed in TFH lymphoma.	5: 61%<br>4: 33%<br>3: 6%<br>2: 0%<br>1: 0%	Consensus reached

**Key Question 7: Which clinical visits and diagnostic tests are required for appropriate follow-up, and how frequently should they be conducted?**		
In cases of a negative end-of-treatment PET scan with evidence of complete response, follow-up can be conducted with CT imaging alone.	5: 83%<br>4: 17%<br>3: 0%<br>2: 0%<br>1: 0%	Consensus reached
Given the marked heterogeneity of the disease, follow-up assessments and their frequency may vary significantly in clinical practice, depending on the initial presentation and disease stage.	5: 83%<br>4: 17%<br>3: 0%<br>2: 0%<br>1: 0%	Consensus reached
In systemic T-cell lymphomas treated with induction therapy, a PET scan is performed to assess response to first-line treatment and to determine the patient's eligibility for ASCT. PET imaging is repeated after transplantation; if the result is negative, a CT scan is performed and subsequently repeated every 4 months for 2 years, along with routine blood tests. Thereafter, CT scans are performed every 6–12 months for an additional 3 years, with blood tests every 3–4 months, completing a total follow-up period of 5 years.	5: 72%<br>4: 17%<br>3: 11%<br>2: 0%<br>1: 0%	Consensus reached
During the first year after treatment, blood tests are recommended every 3–4 months and CT scans every 4–6 months.	5: 89%<br>4: 11%<br>3: 0%<br>2: 0%<br>1: 0%	Consensus reached
**Key Question 8: What salvage treatment options are recommended for relapsed or refractory disease?**		
If a patient with relapsed systemic T-cell lymphoma has not undergone ASCT as part of first-line treatment, autologous transplantation may be considered in combination with salvage therapy and second-line brentuximab.	5: 78%<br>4: 22%<br>3: 0%<br>2: 0%<br>1: 0%	Consensus reached
Relapsed/refractory nodal and extranodal PTCL, except ALK-positive ALCL, ENKTCL		
If no clinical trial is available, platinum-based regimens may be considered.	5: 83%<br>4: 17%<br>3: 0%<br>2: 0%<br>1: 0%	Consensus reached
For patients with relapsed/refractory PTCL-NOS and TFH lymphoma, salvage regimens such as ICE, DHAP, GDP, and IVAC–MTX may be considered; azacitidine is used exclusively for TFH lymphoma.	5: 89%<br>4: 11%<br>3: 0%<br>2: 0%<br>1: 0%	Consensus reached
For patients with relapsed/refractory ALK-negative ALCL and BIA-ALCL, salvage regimens, such as ICE, DHAP, GDP, and IVAC–MTX, may be considered. BV monotherapy may also be an option.	5: 83%<br>4: 17%<br>3: 0%<br>2: 0%<br>1: 0%	Consensus reached
For patients with relapsed/refractory HSTCL and MEITL, ICE and DHAP regimens may be considered.	5: 83%<br>4: 17%<br>3: 0%<br>2: 0%<br>1: 0%	Consensus reached
Treatment options for patients with relapsed/refractory EATL and intestinal T-cell lymphoma-NOS include ICE, DHAP and IVAC–MTX regimens.	5: 83%<br>4: 17%<br>3: 0%<br>2: 0%<br>1: 0%	Consensus reached

For transplant-eligible patients who respond to salvage therapy, HSCT may be considered – autologous HSCT if not used in first-line treatment, allo-HSCT if not previously performed, or, in selected cases, a sequential auto-allo approach.	5: 83%<br>4: 17%<br>3: 0%<br>2: 0%<br>1: 0%	Consensus reached
Relapsed/refractory ALK-positive ALCL		
BV-containing therapy is recommended for patients who have not received BV as part of first-line treatment or for those with a late relapse after an initial response.	5: 78%<br>4: 22%<br>3: 0%<br>2: 0%<br>1: 0%	Consensus reached
Chemotherapy (ICE, DHAP, or IVAC–MTX) may be considered as a potential treatment option.	5: 78%<br>4: 22%<br>3: 0%<br>2: 0%<br>1: 0%	Consensus reached
Autologous transplantation should be considered as a consolidation therapy, based on response to salvage treatment, the quality of current remission, comorbidities, and anticipated tolerability.	5: 72%<br>4: 28%<br>3: 0%<br>2: 0%<br>1: 0%	Consensus reached
Relapsed/refractory ENKTCL		
Gemcitabine and/or L-asparaginase-based cycles may be used. As an alternative, platinum-based regimens (e.g., GDP) can be considered.	5: 78%<br>4: 22%<br>3: 0%<br>2: 0%<br>1: 0%	Consensus reached
For transplant-eligible patients who respond to salvage therapy, SCT may be considered—preferably allo-HSCT if not used in first-line treatment.	5: 83%<br>4: 17%<br>3: 0%<br>2: 0%<br>1: 0%	Consensus reached
Relapsed/refractory T-LGL and NK-LGL		
An alternative immunosuppressive agent among those recommended for first-line treatment may be considered (MTX, cyclophosphamide, cyclosporine A).	5: 72%<br>4: 28%<br>3: 0%<br>2: 0%<br>1: 0%	Consensus reached
For transplant-eligible patients who respond to salvage therapy, SCT may be considered – preferably allo-HSCT if not previously used in first-line treatment.	5: 78%<br>4: 22%<br>3: 0%<br>2: 0%<br>1: 0%	Consensus reached
Relapsed/refractory ATLL		
Second-line therapy with a platinum-based regimen.	5: 78%<br>4: 22%<br>3: 0%<br>2: 0%<br>1: 0%	Consensus reached
For transplant-eligible patients who respond to salvage therapy, SCT may be considered – preferably allo-HSCT if not used in first-line treatment.	5: 76%<br>4: 24%<br>3: 0%<br>2: 0%<br>1: 0%	Consensus reached

Consensus was defined based on a predefined agreement threshold (≥75%), calculated by summing the percentages of responses scoring 4 (agreement) and 5 (full agreement), as indicated by participants on a 5-

point Likert scale (1 = complete disagreement; 5 = complete agreement).
AITL: angioimmunoblastic-type T-cell lymphoma; ALCL: anaplastic large cell lymphoma; allo-HSCT: allogeneic hematopoietic stem cell transplantation; allo-SCT: allogeneic stem cell transplantation; ASCT: autologous stem cell transplant; HSCTL: Hematopoietic Stem Cell Transplantation – Leukemia; AspMetDex: PEG-Asp, methotrexate, and dexamethasone; ATLL: adult T-cell leukemia/lymphoma; BMB: bone marrow biopsy; BV-CHP: Brentuximab vedotin – Cyclophosphamide – Hydroxydaunorubicin - Prednisone; CHOEP: cyclophosphamide, Hydroxydaunorubicin, Oncovin, etoposide and prednisone; CHOP: cyclophosphamide, doxorubicin, vincristine, and prednisone; CVAD: cyclophosphamide, vincristine, doxorubicin, and dexamethasone; DDGP: cisplatin, dexamethasone, gemcitabine, and pegaspargase; DHAOx: dexamethasone, high-dose cytarabine, and oxaliplatin; DHAP: dexamethasone, high-dose cytarabine and cisplatin; DHAX: dexamethasone, cytarabine, and oxaliplatin, EATL: enteropathy-associated T-cell lymphoma; ENKTCL: extranodal NK-/T-cell lymphoma; EPOCH: EPOCH-R: etoposide, prednisone, vincristine, cyclophosphamide, doxorubicin; FISH: fluorescence in situ hybridization; GDP: gemcitabine, dexamethasone, and cisplatin; GEMOX: gemcitabine and oxaliplatin; HSCT: hematopoietic stem cell transplantation; ICE: ifosfamide, carboplatin and etoposide; IPI: International Prognostic Index; IVAC: ifosfamide, etoposide, cytarabine; IVE: ifosfamide, epirubicin, and etoposide; MEITL: monomorphic epitheliotropic intestinal t-cell lymphoma; mSMILE: modified – **S**teroids – **M**ethotrexate – **I**fosfamide - **L**-Asparaginasi - Etoposide; MTX: methotrexate; NK-LGL: natural killer large granular lymphocytic leukemia; P-GEMOX: pegaspargase, gemcitabine and oxaliplatin; PINK: Prognostic Index of Natural Killer lymphoma; PINK-E: Prognostic Index of extranodal Natural Killer lymphoma; PIT: Prognostic Index for PTCL-Unspecified; PTCL: peripheral T-cell lymphoma; PTCL-NOS: peripheral T-cell lymphoma not otherwise specified; RT: radiotherapy; SCT: stem cell transplantation; TFH: T follicular helper; T-LGL: T-cell large granular lymphocytic leukemia.

Supplementary Table 6. Breakdown of panelists

**Author**	**Affiliation**	**Region**	**Specialty**	**Working Group Assignment**
Attilio Guarini	Chief of the Hematology Unit, IRCCS Istituto Tumori "Giovanni Paolo II", Bari, Italy	Puglia	Hematology	Mantle Cell Lymphoma
Valentina Bozzoli	UO Ematologia e Trapianto di Cellule Staminali, Ospedale Vito Fazzi, asl Lecce, Italy	Puglia	Hematology	DLBCL
Sabino Ciavarella	Lymphoma Unit - Hematology Unit, IRCCS Istituto Tumori "Giovanni Paolo II", Bari, Italy	Puglia	Hematology	Mantle Cell Lymphoma
Michele Cimminiello	SIC di Ematologia con TMO, AOR San Carlo di Potenza, Potenza, Italy	Basilicata	Hematology	Peripheral T-cell lymphomas
Francesca Donatelli	UOC Ematologia e Trapianto di CSE azienda ospedaliera C.Panico Tricase, Lecce, Italy	Puglia	Hematology	DLBCL
Angelo Fama	UOS Ematologia, Dipartimento di Medicina Interna e Specialità Mediche, Ospedale "Giuseppe Mazzini" Hospital, ASL Teramo, 64100 Teramo, Italy	Abruzzo	Hematology	Follicular lymphoma
Vincenza Fernanda Fesce	UO Ematologia con Trapianto di CSE Azienda Ospedaliero, Universitaria Policlinico Riuniti di Foggia, Foggia, Italy	Puglia	Hematology	Peripheral T-cell lymphomas
Vincenzo Fraticelli	Unità Operativa Semplice a valenza Dipartimentale di Onco-Ematologia Largo Gemelli n°1 86100, Campobasso, Italy	Molise	Hematology	Follicular lymphoma
Francesco Gaudio	Chief of the Unit of Hematology, “F. Miulli” University Hospital, Acquaviva delle Fonti, Bari, Italy | Department of Medicine and Surgery, LUM University “Giuseppe Degennaro”, Casamassima-Bari, Italy	Puglia	Hematology	Classical Hodgkin lymphoma
Giuseppina Greco	UOC Ematologia e Trapianto di CSE azienda ospedaliera C.Panico Tricase, Lecce, Italy	Puglia	Hematology	DLBCL

Martellini Augusto	Polistudium SRL, Milan, Italy	Lombardia	Medical Communicatio n	-
Francesca Merchionne	Lymphoma Unit - U.O. Ematologia Ospedale "Antonio Perrino", 72100, Brindisi, Italy	Puglia	Hematology	Mantle Cell Lymphoma
Rosanna Maria Miccolis	UOC Ematologia con Trapianto P.O. "Mons.Dimiccoli" Barletta, Italy	Puglia	Hematology	Classical Hodgkin lymphoma
Carla Minoia	Lymphoma Unit - Hematology Unit, IRCCS Istituto Tumori "Giovanni Paolo II", Bari, Italy	Puglia	Hematology	Follicular lymphoma
Elsa Pennese	Head of the lymphoma Unit - UOC Ematologia Clinica Dipartimento Oncologico Ematologico Presidio Ospedaliero Spirito Santo, Pescara, Italy	Abruzzo	Hematology	DLBCL
Tommasina Perrone	Lymphoma Unit - Hematology and Stem Cells Transplantation, AOUC Policlinico, Bari, Italy	Puglia	Hematology	Mantle Cell Lymphoma
Potito Rosario Scalzulli	Lymphoma Unit - UOC Ematologia e Trapianto di Cellule Staminali Emopoietiche, Fondazione IRCCS "Casa Sollievo della Sofferenza", San Giovanni Rotondo (FG), Italy	Puglia	Hematology	Classical Hodgkin lymphoma
Anna Scattone	Pathology Unit, IRCCS Istituto Tumori "Giovanni Paolo II", Bari, Italy	Puglia	Hemolympho-Pathology	Follicular lymphoma, DLBCL, CHL, MCL, PTCL
Tetiana Skrypets	Lymphoma Unit - Hematology Unit, IRCCS Istituto Tumori "Giovanni Paolo II", Bari, Italy	Puglia	Hematology	Peripheral T-cell lymphomas
Mariarosaria Specchia	S.C. Ematologia e Trapianto di CSE ospedale “S. G. Moscati” ASL Taranto, Italy	Puglia	Hematology	DLBCL
Lorenzo Tonialini	Lymphoma Unit - Hematology Unit, IRCCS Istituto Tumori "Giovanni Paolo II", Bari, Italy	Puglia	Hematology	Classical Hodgkin lymphoma
Mariarosaria Valvano	UOC Ematologia e Trapianto di Cellule Staminali Emopoietiche, Fondazione IRCCS "Casa Sollievo della	Puglia	Hematology	Classical Hodgkin lymphoma

	Sofferenza", San Giovanni Rotondo (FG), Italy			
Vincenzo Pavone	Past Chief of the UOC Ematologia e Trapianto di CSE azienda ospedaliera C.Panico Tricase, Lecce, Italy	Puglia	Hematology	DLBCL

Supplementary Note: Evidence Review Methodology

To support the development of the consensus statements, a focused literature review was conducted by the coordinating team and scientific board to ensure alignment with the most current clinical evidence. The review was not systematic but followed a structured approach consistent with scoping methodology.

Sources Consulted

- **PubMed/MEDLINE** database (latest search: March 2025)
- International guideline repositories:
    - European Society for Medical Oncology (ESMO)
    - National Comprehensive Cancer Network (NCCN)
    - Fondazione Italiana Linfomi (FIL)
- 2022 WHO Classification and 2022 International Consensus Classification (ICC)

Inclusion Criteria

- Publications from **January 2018 onward**
- High-impact studies relevant to key clinical decisions addressed in the consensus (e.g., diagnostic strategies, first-line and salvage therapies, imaging and follow-up)
- Randomized controlled trials, meta-analyses, international guidelines, and pivotal phase II/III studies
- Studies specific to the five lymphoma subtypes discussed: cHL, DLBCL, FL, MCL, PTCL

Selection Strategy

The scientific board prioritized studies based on clinical relevance, level of evidence, and applicability to the Italian healthcare context. When available, recent multicenter trials and European or global consensus papers were favored. Expert opinion was used to complement areas with limited data (e.g., rare subtypes, follow-up strategies).

This review process informed the refinement of statements during the Nominal Group Technique and Delphi rounds and supported the application of simplified evidence levels (A/B/C) in Supplementary Table 1.
